# Genetic Evidence for the Role of the Vacuole in Supplying Secretory Organelles with Ca^2+^ in *Hansenula polymorpha*

**DOI:** 10.1371/journal.pone.0145915

**Published:** 2015-12-30

**Authors:** Anastasia V. Fokina, Maria B. Chechenova, Azamat V. Karginov, Michael D. Ter-Avanesyan, Michael O. Agaphonov

**Affiliations:** A.N. Bach Institute of Biochemistry, Research Center of Biotechnology of the Russian Academy of Sciences, Moscow, Russia; University of Cambridge, UNITED KINGDOM

## Abstract

Processes taking place in the secretory organelles require Ca^2+^ and Mn^2+^, which in yeast are supplied by the Pmr1 ion pump. Here we observed that in the yeast *Hansenula polymorpha* Ca^2+^ deficiency in the secretory pathway caused by Pmr1 inactivation is exacerbated by (i) the *ret1-27* mutation affecting COPI-mediated vesicular transport, (ii) inactivation of the vacuolar Ca^2+^ ATPase Pmc1 and (iii) inactivation of Vps35, which is a component of the retromer complex responsible for protein transport between the vacuole and secretory organelles. The *ret1-27* mutation also exerted phenotypes indicating alterations in transport between the vacuole and secretory organelles. These data indicate that *ret1-27*, *pmc1* and *vps35* affect a previously unknown Pmr1-independent route of the Ca^2+^ delivery to the secretory pathway. We also observed that the vacuolar protein carboxypeptidase Y receives additional modifications of its glycoside chains if it escapes the Vps10-dependent sorting to the vacuole.

## Introduction

A number of processes in the secretory pathway of eukaryotes require the presence of Ca^2+^ and Mn^2+^. The Golgi apparatus is supplied with these ions by means of the secretory pathway calcium ATPase. In yeast this protein is encoded by the *PMR1* gene [1]. The endoplasmic reticulum (ER) of animal cells is a Ca^2+^ storage organelle, which participates in signal transduction by releasing Ca^2+^ to the cytosol. Its Ca^2+^ pool is replenished by a special ion pump, the sarco/endoplasmic reticulum Ca^2+^ ATPase. In yeast, the ER is probably not involved in the Ca^2+^ signaling and no ER Ca^2+^ pump has been identified. However the processes taking place in the ER lumen, which are related to protein secretion, still require Ca^2+^. In yeast, the Golgi apparatus Ca^2+^ ATPase is a major contributor to the ER supply of Ca^2+^, since inactivation of the *Saccharomyces cerevisiae PMR1* gene leads to a 50% decrease in the ER Ca^2+^ level [2]. However the source of the ER Ca^2+^ in the absence of Pmr1 remains unknown.

The main yeast Ca^2+^ storage/sink organelle is the vacuole, which possesses its own Ca^2+^ATPase Pmc1 and the H^+^/Ca^2+^ antiporter Vcx1. These proteins serve to replenish the vacuolar Ca^2+^ pool and to maintain cytosolic Ca^2+^ concentration at low levels [3, 4, 5]. In *S*. *cerevisiae* inactivation of the *PMC1* gene leads to sensitivity to high concentrations of Ca^2+^ in culture medium and is lethal in absence of the *PMR1* gene. Both these mutant phenotypes are suppressed by inactivation of the Ca^2+^/calmodulin-dependent protein phosphatase calcineurin, indicating that increased Ca^2+^ concentration blocks cell growth due to calcineurin activation, while Pmr1 acts together with Pmc1 in maintaining the cytosolic Ca^2+^ concentration at a low level [3].

Many vacuolar proteins are synthesized in the ER together with secretory proteins. Then they are sorted at the late Golgi to be delivered to the vacuole by vesicular transport via different routes [6]. For example, carboxypeptidase Y (CPY) is sorted to the vacuole by the Vps10 receptor, which cycles between the late Golgi and the endosomal or prevacuolar compartments [7, 8]. Proteins endocytosed from the cell surface are also transported to the vacuole [9, 10, 11, 12, 13, 14], while some secretory proteins pass through the endosomal compartments prior to exocytosis [15]. These pathways require intensive anterograde and retrograde traffic of proteins and lipids between the vacuole, endosomes and secretory compartments. Thus it is possible that this traffic connects the vacuolar Ca^2+^ pool to the secretory organelles.

Retrograde transport of proteins and lipids through the secretory organelles is mediated by COPI coated vesicles. The COPI coat consists of α, β, ε, β', γ, δ, and ζ subunits. The former three subunits comprise subcomplex B; the latter four comprise subcomplex F [16, 17]. Previously we have shown that in the methylotrophic yeast *Hansenula polymorpha* C-terminal truncation of α-COP leads to sensitivity to Ca^2+^ shortage in the culture medium [18]. This indicates involvement of the COPI coated vesicles in Ca^2+^ trafficking in the cell. Besides, COPI subunits were shown to be involved in membrane traffic in the endocytic pathway in animal cells [19, 20, 21]. In yeast, mutations in the components of the COPI subcomplex B were shown to affect protein sorting from late endosomes to the vacuole [22].

In this work we have obtained data indicating involvement of the vacuole in supply of the secretory pathway with Ca^2+^, which can be mediated by COPI-dependent vesicular transport.

## Materials and Methods

### Culture conditions and genetic methods

*H*. *polymorpha* cells were cultivated at 37°C in complex YPD (1% yeast extract, 2% peptone, 2% glucose) and YPM (1% yeast extract, 2% peptone and 1% methanol) media or in synthetic SD medium (0.67% Yeast Nitrogene Base (Difco), 2% glucose) and Ca^2+^-deficient medium [1] SD* (Ca^2+^-deficient Yeast Nitrogen Base, 2% glucose). Solid media contained 2,5% agar. The Ca^2+^-deficient Yeast Nitrogen Base was made from 5 separately prepared solutions: A (ammonium sulfate 500 g/L), B (biotin 0.2 mg/L, folic acid 0.2 mg/L, inositol 2 g/L, niacin 0.4 g/L, pyridoxine hydrochloride 0.4 g/L, riboflavin 0.2 g/L, thiamine hydrochloride 0.4 g/L, calcium pantothenate 0.4 g/L, p-aminobenzoic acid 0.2 g/L), C (KH_2_PO_4_ 20 g/L, MgSO_4_ 10 g/L, NaCl 2 g/L), D (boric acid 5 g/L, CuSO_4_·5H_2_O 0.4 g/L, KI 0.5 g/L, FeCl_3_ 2 g/L, MnCl_2_·4H_2_O 4 g/L, ZnSO_4_ 4 g/L), D' (Na_2_MoO_4_·2H_2_O 2 g/L). To obtain the Ca^2+^-deficient Yeast Nitrogen Base 10X solution, components A, B, C, D, D' and deionized H_2_O were mixed in proportion 10:10:50:0.01:0.01:30. Additional depletion of Ca^2+^ was achieved by supplementing the SD* with ethylene glycol tetraacetic acid (EGTA). Where required, leucine, adenine, or uracil were added. *H*. *polymorpha* strains were crossed, and hybrids were sporulated on maltose-containing medium (2% maltose, 3% agar). For induction of expression of the unglycosylated mutant of urokinase-type plasminogen activator (uPA-Q^302^), overnight cultures grown in liquid YPD containing 0.1 M NaCl were diluted six-fold with induction medium containing 1% yeast extract, 3% peptone 25 mM NH_4_H_2_PO_4_, 25 mM (NH_4_)_2_HPO_4_, 0,1 M NaCl, 0.05% glycerol, 0.8% MeOH and incubated at 37°C for 70 h. For the analysis of CPY secretion cells were grown in liquid YPD for 40 h. *H*. *polymorpha* was transformed according to the modified lithium acetate method [23].

### Strains

Strains used in this study are listed in Table 1. All the strains originated from the *H*. *polymorpha* CBS4732 (*Ogataea polymorpha*). The 64MA70 strain was obtained as a haploid segregant from the cross between the strains 1B (*leu2 ade2*) [23] and 2dMA56 (*leu2 ade2 ret1-27 mox*::*uPA*) [18]. To obtain 64MA70U and 64MA70Q, the *MOX* gene in the 64MA70 strain was replaced with either uPA or uPA-Q^302^ expression cassettes, respectively, as described [24]. The derivatives of these strains, which were disrupted for *VPS10*, *VPS35*, or *PMC1*, were obtained by transformation with disruption cassettes excised from the pU15, pCAF11, or pKAF2 plasmids, respectively. The derivatives with the wild type *RET1* allele were obtained by integration of the p2CHA6-27OPU plasmid into genome. If the derivative strains possessed the *leu2* or *ade2* mutations, plasmids pCHLX or pCHAD3 bearing *LEU2* or *ADE2*, respectively, were introduced into their genomes to obtain prototrophic strains.

**Table 1:** *H*. *polymorpha* strains.

Strain	Genotype	Reference
1MA77/12/p2CHA6	*leu2 ade2 mox*::*uPA-Q* ^*302*^ *pmr1*::*LEU2 [ADE2]*	[25]
1MA77/12/GP1-Δvps35	*leu2 ade2 mox*::*uPA-Q* ^*302*^ *pmr1*::*G418 vps35*::*LEU2 [PMR1 ADE2]*	This study
1MA77/12/GAP2-Δpmc	*leu2 ade2 mox*::*uPA-Q* ^*302*^ *ura3*::*ADE2 pmr1*::*G418 pmc1*::*loxP [URA3 PMR1]*	This study
1MA77/12/GAP2	*leu2 ade2 mox*::*uPA-Q* ^*302*^ *ura3*::*ADE2 pmr1*::*G418 [URA3 PMR1]*	This study
1MA77/12/GP1	*leu2 ade2 mox*::*uPA-Q* ^*302*^ *pmr1*::*G418 [PMR1 ADE2]*	[25]
64MA70Q-RET-Δpmc	*leu2 ade2 ret1-27 mox*::*uPA-Q* ^*302*^ *pmc1*::*LEU2 [RET1 ADE2]*	This study
64MA70QL-RET	*leu2 ade2 ret1-27 mox*::*uPA-Q* ^*302*^ *[LEU2] [RET1 ADE2]*	This study
64MA70QA-Δpmc	*leu2 ade2 ret1-27 mox*::*uPA-Q* ^*302*^ *pmc1*::*LEU2 [ADE2]*	This study
64MA70Q-RET-Δvps10	*leu2 ade2 ret1-27 mox*::*uPA-Q* ^*302*^ *vps10*::*LEU2 [RET1 ADE2]*	This study
64MA70QAL	*leu2 ade2 ret1-27 mox*::*uPA-Q* ^*302*^ *[LEU2] [ADE2]*	This study
64MA70U-RET-Δvps10	*leu2 ade2 ret1-27 mox*::*uPA vps10*::*LEU2 [RET1 ADE2]*	This study
64MA70U-RET-Δvps35	*leu2 ade2 ret1-27 mox*::*uPA vps35*::*LEU2 [RET1 ADE2]*	This study
64MA70UA-RET	*leu2 ade2 ret1-27 mox*::*uPA [RET1 LEU2] [ADE2]*	This study
64MA70UA-Δvps10	*leu2 ade2 ret1-27 mox*::*uPA vps10*::*LEU2 [ADE2]*	This study
64MA70UA-Δvps35	*leu2 ade2 ret1-27 mox*::*uPA vps35*::*LEU2 [ADE2]*	This study
64MA70UAL	*leu2 ade2 ret1-27 mox*::*uPA [LEU2] [ADE2]*	This study
MC39	*leu2 ade2 ret1-27 pmr1*::*LEU2 [PMR1 ADE2]*	This study
MC39-MOX	*leu2 ade2 pmr1*::*LEU2 ret1-27 mox*::*uPA-Q* ^*302*^ *[PMR1 ADE2] [MOX]*	This study
MC39-RET-MOX	*leu2 ade2 pmr1*::*LEU2 ret1-27 mox*::*uPA-Q* ^*302*^ *[PMR1 ADE2] [MOX RET1]*	This study

The *leu2* auxotrophic marker in the *pmr1-Δ* strain 1MA77/12 (*leu2 ade2 mox*::*uPA pmr1*::*LEU2 [PMR1 ADE2]*) was restored by replacing the *pmr1*::*LEU2* allele with the *pmr1*::*G418* ^*r*^ allele from the pAF14 plasmid as described [25]. The resulting 1MA77/12/GP1 strain was disrupted for the *VPS35* gene using the pCAF11-derived disruption cassette to obtain the 1MA77/12/GP1-Δvps35 strain. The 1MA77/12/GAP2 strain was constructed by disruption of the *URA3* gene with the *ADE2* selectable marker in the 1MA77/12/GP1 strain and by subsequent introduction of the autonomously replicating plasmid pAF18 bearing the *URA3* selectable marker and the *PMR1* gene. The 1MA77/12/GAP2-Δpmc1 strain was obtained by disruption of the *PMC1* gene in 1MA77/12/GAP2 using the pAM655 plasmid. The MC39 strain (*leu2 ade2 mox*::*u-PAQ* ^*302*^ *ret1-27 pmr1*::*LEU2 [PMR1*, *ADE2]*) is a segregant from the cross between the *ret1-27* mutant 2dMA56 and the *pmr1-Δ* mutant 1MA77/12. Its derivatives MC39-MOX and MC39-RET-MOX were obtained by introduction of the pMOX-H36 or pRET1-MOX plasmid, respectively.

### Plasmids

Plasmids used are listed in Table 2. The p2CHA6-27OPU plasmid was constructed by insertion of the *Bam*HI-*Ehe*I fragment of p27OPU8 [18], which carries the *RET1* gene with its native promoter, into the p2CHA6 vector [25]. Although this plasmid is capable of autonomous replication in *H*. *polymorpha* cells, it was always digested with *Bam*HI and *Eco*RI prior to transformation to achieve genomic integration of its fragment bearing the *ADE2* and *RET1* genes. pAF18 was constructed by insertion of the *Sac*I-*Eco*RI fragment of pE1, which carries the *PMR1* gene with its native promoter, between the *Sac*I and *Eco*RI restriction sites of the pAM459 vector. The latter vector was obtained from the pRS426 *S*. *cerevisiae* shuttle vector [26] by *in vivo* capturing of DNA fragment improving its autonomous maintenance in *H*. *polymorpha* cells as follows. The *H*. *polymorpha ura3* mutant was transformed with pRS426 digested with *Nde*I and *Sna*BI. The digestion was aimed to induce random capturing of a DNA fragment, which would improve the plasmid replication and the selectable marker expression like it was described previously [23]. Finally, the plasmid, which was designated as pAM459, was isolated from one of the obtained transformants. The pCHAD3 plasmid was obtained by cloning of the *H*. *polymorpha ADE2* gene into the pBC-SK(+) vector. To construct pCAF11, the *VPS35* gene was amplified by PCR and its Eco47III-SalI fragment was cloned in the pBC-KS(+) vector. Then the *Bam*HI-*Eco*RI fragment within the *VPS35* ORF was replaced with the *LEU2*-carrying *Bam*HI-*Eco*RI fragment of the pCHLX plasmid. Prior to yeast transformation, pCAF11 was digested with *Bgl*II and *Sal*I.

**Table 2:** Plasmids.

Plasmid	Functional elements	Reference
p2CHA6-27OPU	Wild-type *RET1* gene and the *ADE2* selectable marker	This study
pAF14	*PMR1* disruption cassette with G418 resistance marker	[25]
pAF18	Wild-type *PMR1* gene, the *S*.* cerevisiae URA3* as a selectable marker and 2μ DNA fragment for maintenance in the autonomous state.	This study
pAHAD1	*ADE2* selectable marker	This study
pAM655	The self-excising *PMС1* disruption cassette with *LEU2* selectable marker	[27]
pCAF11	*VPS35* disruption cassette with *LEU2* selectable marker	This study
pCHAD3	*ADE2* selectable marker	This study
pCHLX	*LEU2* selectable marker	[28]
pKAF2	*PMС1* disruption cassette with modified *S*.* cerevisiae LEU2* as a selectable marker	[29]
pMOX-H36	*MOX* gene as a selectable marker and autonomously replicating sequence	This study
pRET1-MOX	Wild-type *RET1* gene and the *MOX* gene as a selectable marker	This study
pU15	*VPS10* disruption cassette with *LEU2* selectable marker	[30]

The pMOX-H36 plasmid contained the *Sph*I-*Ngo*MI fragment of *H*. *polymorpha* genomic DNA bearing the *MOX* gene and the *Hin*dIII-*Sph*I fragment of AMIpSL1, possessing *HARS36* [31]. These two fragments were inserted between the *Hin*dIII and *Xma*I sites of the pTZ19R vector. pRET1-MOX was obtained by replacement of the *HARS36*-bearing fragment of the pMOX-H36 plasmid with the *Ehe*I-*Sph*I fragment of p27OPU8, bearing the *RET1* gene with its native promoter.

### Electrophoresis and immunoblotting

Proteins from culture supernatants were concentrated 3-fold in case of CPY analysis and 20-fold in the case of uPA and uPA-Q^302^ analysis by precipitation with trichloroacetic acid. The amounts of uPA, uPA-Q^302^ and CPY from culture medium and in cell lysates were normalized to the levels of total cellular protein as described in [32] and subsequently resolved by electrophoresis in 10% polyacrylamide gel as described in [33]. Rabbit antisera against *E*. *coli* expressed *H*. *polymorpha* CPY [30] and Pmr1 [18], tubulin beta antibody (PA5-16863, ThermoFisher Scientific) and monoclonal mouse antibody specific to the uPA protease domain (#MGH U11, IMTEK, Moscow, Russia) were used to detect corresponding proteins. The immunoblots were developed using SuperSignal West Dura Extended Duration Substrate (Thermo Scientific, Illinois, U.S.A.).

### Analysis of carboxypeptidase Y proteolytic fragments

Two major forms of CPY were revealed in cell lysates and culture supernatants of the *H*. *polymorpha vps35-Δ* and *vps10-Δ* mutants. After treatment with endoglycosidase H_f_ (EndoH, New England BioLabs, Ipswich, Massachusetts, U.S.A.), one of them migrated close to the 46 kDa marker band, the other—between the 46 kDa and 30 kDa marker bands. CPY from culture supernatants was partially purified by ion exchange chromatography, treated with EndoH and separated by SDS PAGE. Gel fragments containing CPY were prepared, incubated with sequencing grade trypsin (Promega, Madison, Wisconsin, U.S.A.), and the peptides were analyzed using an Ultraflextreme MALDI-TOF/TOF mass spectrometer (Bruker Daltonics, Billerica, Massachusetts, U.S.A.) equipped with an Nd laser (354 nm) as described elsewhere [34]. According to this analysis both CPY forms possessed an intact C-terminus and were shortened at the N-terminus. The larger form lacked only the pro-region (up to K^122^), which was predicted based on homology to the *S*. *cerevisiae* ortholog [35]. Calculated molecular weight of the *H*. *polymorpha* CPY polypeptide chain starting from K123 is 47 kDa. In the shorter form, the most N-terminal peptide, which was revealed by mass spectrometric analysis, started from S^227^.

### Analysis of vacuolar morphology

Cells grown in liquid YPD were collected at exponential phase, washed with H_2_O, and then with 10 mM HEPES buffer pH 7.4, containing 5% D-glucose, resuspended at a density of 10^6^ cells/ml in the same HEPES buffer, containing glucose and 200 μM CellTracker Blue CMAC (Thermo Scientific). Cells were incubated for 30–45 min at room temperature and visualized by fluorescence microscopy using an Axioskop 40 (Zeiss, Oberkochen, Germany) with a cooled CCD camera (Olympus Corporation, Tokyo, Japan). Images were assembled in Photoshop (Adobe) with only linear adjustments.

## Results

### A defect of COPI-mediated vesicular transport exacerbates the requirement of *H*. *polymorpha pmr1-Δ* mutant for Ca^2+^ and Mn^2+^

The mutant *ret1-27* allele encodes α-COP lacking more than 300 C-terminal amino acids. Its manifestations, e.g. inability to grow on Ca^2+^-depleted medium and enhanced ability to secrete human uPA, resemble those of the *pmr1-Δ* mutation, which inactivates the Golgi apparatus Ca^2+^ ATPase [18, 25]. It was previously suggested that these phenotypes of *ret1-27* are due to the decreased function of Pmr1 [18]. Here, to ascertain whether the effect of *ret1-27* on the Ca^2+^ homeostasis is indeed mediated by Pmr1, we studied interaction between the *pmr1-Δ* and *ret1-27* mutations. To do this we used the strain MC39 carrying these mutations and the autonomously-replicating plasmid with the *PMR1* gene (see Materials and Methods and Table 1). Cells of this strain were able to lose the *PMR1*-carrying plasmid only after introduction of the second plasmid bearing the wild type *RET1* gene, which indicated that the *ret1-27* and *pmr1-Δ* mutations were synthetically lethal. Remarkably, cells of the MC39 strain grown on medium supplemented with 10 mM CaCl_2_ could lose the *PMR1*-containing plasmid even in the absence of the *RET1* plasmid, indicating that synthetic lethality was caused by insufficient supply of the secretory organelles with Ca^2+^. Transferring cells of the *pmr1-Δ ret1-27* double mutant to regular YPD medium led to rapid cell death accompanied by DNA fragmentation (S1 Fig), resembling what was previously observed in a strain carrying the *pmr1-Δ* mutation alone upon incubation in medium with phosphate buffer and methanol as a sole carbon source [25]. Thus the *ret1-27* mutation exacerbated this effect, since in the *pmr1-Δ ret1-27* double mutant, cell death and DNA fragmentation occurred in regular YPD medium.

**Fig 1:**
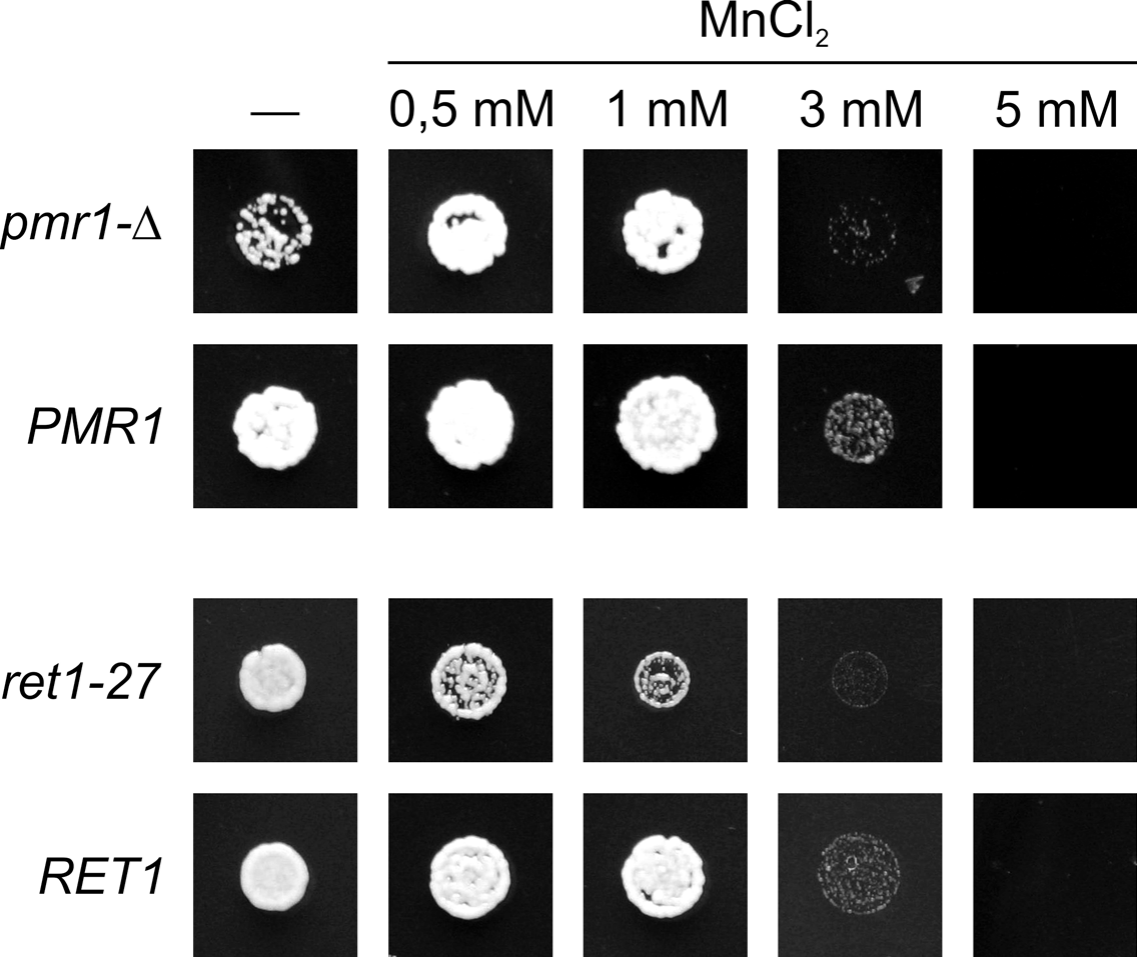
Growth of the *pmr1-Δ* and *ret1-27* mutants on SD* medium supplemented with different concentrations of MnCl_2_. Cell suspensions with equal densities were spotted onto corresponding media and grown for 2 days. The experiment was performed using serial dilutions of cell suspensions (S3 Fig) and a representative dilution is shown in this figure. *pmr1-Δ*, a subclone of the 1MA27/12/GP1 strain lacking the *PMR1* containing plasmid; the *PMR1*, 1MA27/12/GP1 strain, *ret1-27*, the 64MA70QAL strain; *RET1*, the 64MA70QA-RET strain.

**Fig 2:**
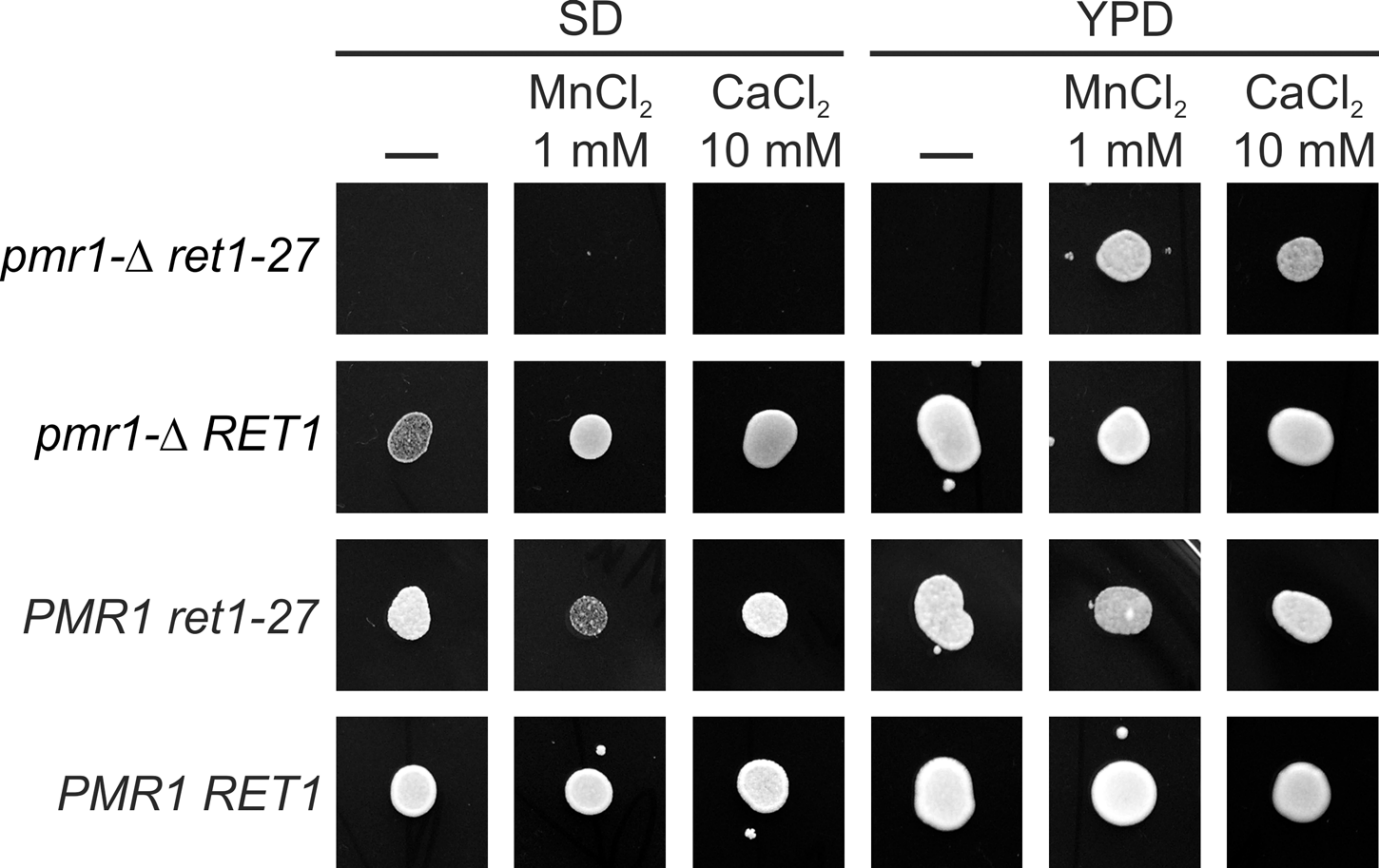
Rescue of growth of the *pmr1-Δ ret1-27* double mutant by CaCl_2_ and MnCl_2_. Since the *ret1-27 pmr1-Δ* double mutant was inviable on regular media, it was obtained from the MC39-MOX strain, which carried a *PMR1-*contaning plasmid. To allow the MC39-MOX and MC39-RET-MOX strains to lose the *PMR1*-contaning plasmid, they were streaked on YPD plate supplemented with 10 mM CaCl_2_. Equal amounts of cells from single colonies obtained on this medium were suspended in sterile water and spotted onto test plates. Growth of only one subclone for each case is shown in the figure. Growth of three additional subclones is shown in S4 Fig *pmr1-Δ ret1-27*, a MC39-MOX subclone lacking the plasmid; *PMR1 ret1-27*, a MC39-MOX subclone retaining the plasmid; *pmr1-Δ RET1*, a MC39-RET-MOX subclone lacking the plasmid; *PMR1 RET1*, a MC39-RET-MOX subclone retaining the plasmid.

In *S*. *cerevisiae* Mn^2+^ was shown to have a dual effect: its cytosolic accumulation is toxic, while it can functionally replace Ca^2+^ in some life essential process(es) and supports cell growth upon Ca^2+^ shortage [36]. Cytosolic accumulation of Mn^2+^ apparently is also toxic in *H*. *polymorpha*, since this yeast was sensitive to elevation of Mn^2+^ concentration in culture medium (Fig 1). Notably, the sensitivity to Mn^2+^ greatly depended on the medium used. The highest sensitivity was observed in SD*, which contains Ca^2+^ in low concentration and is not favorable for growth of the *pmr1-Δ* mutant (S2 Fig). In contrast to the wild-type control strain, the *pmr1-Δ* mutant was almost unable to grow on SD* supplemented with 3 mM MnCl_2_, while 0.5 mM and 1 mM MnCl_2_ noticeably improved its growth (Fig 1). This agrees with the role of Pmr1 in sequestration of Mn^2+^ into the secretory organelles [37]. Remarkably, the *ret1-27* mutation alone also conferred hypersensitivity to Mn^2+^ (Fig 1). At the same time it exacerbated the requirement in external Mn^2+^ caused by the *pmr1-Δ* mutation, since growth of the *ret1-27 pmr1-Δ* double mutant on YPD (but not on SD) could be rescued by elevation of Mn^2+^ concentration and this strain could grow without the *PMR1*-containing plasmid not only in excess of Ca^2+^, but also in the presence of 1 mM MnCl_2_ (Fig 2).

Thus, the *ret1-27* mutation essentially increased the requirement of the *pmr1-Δ* mutant for Ca^2+^ and Mn^2+^. The obtained data demonstrate that the *ret1-27* mutation is able to affect Ca^2+^ homeostasis independently of Pmr1.

### Inactivation of *PMC1* or *VPS35* exacerbates deficiency of Ca^2+^ in the secretory pathway of the *pmr1-Δ* mutant

**Fig 3:**
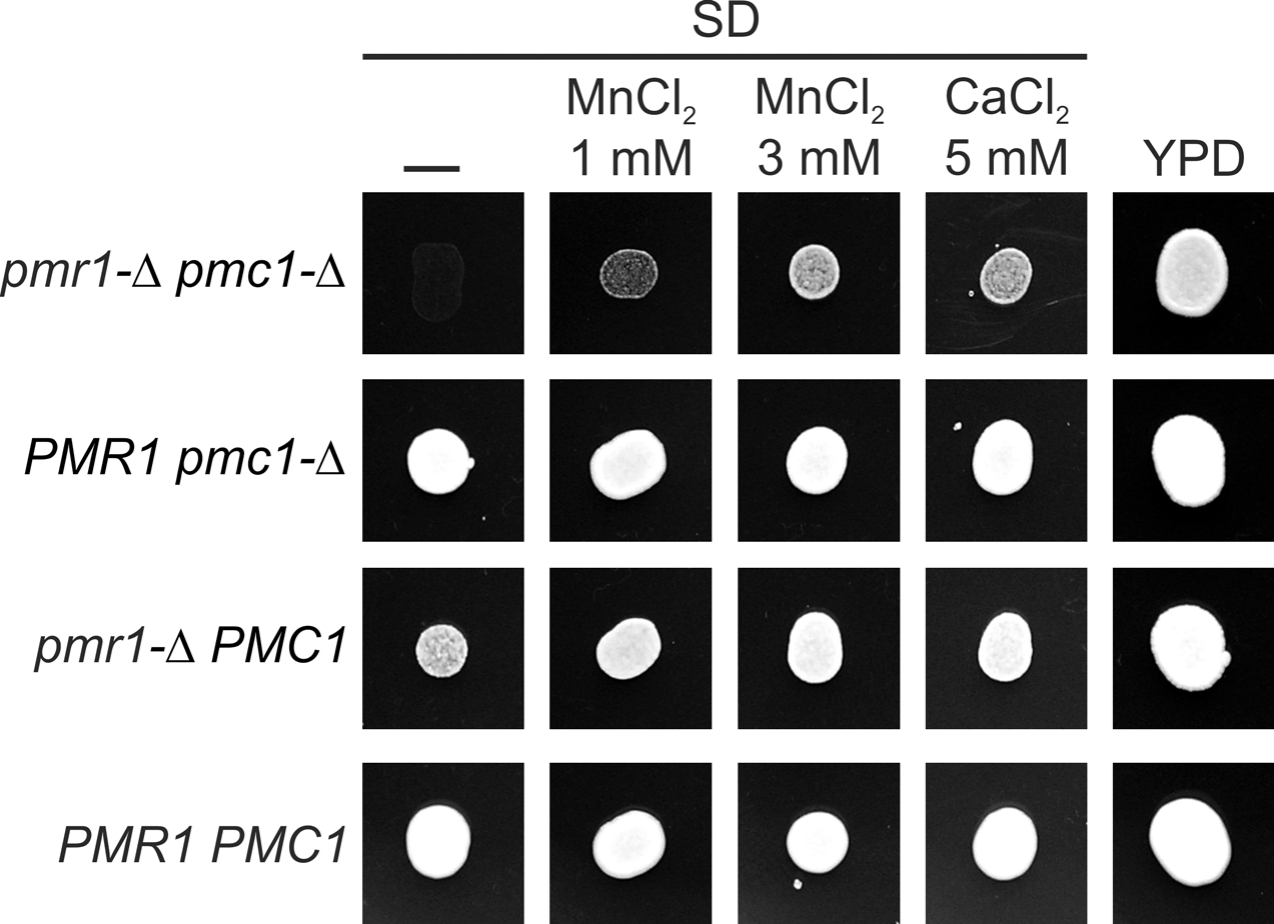
Rescue of growth of the *pmr1-Δ pmc1-Δ* double mutant by CaCl_2_ and MnCl_2_. Cell suspensions with equal densities were spotted onto corresponding media and grown for 2 days. The experiment was performed using serial dilutions of cell suspensions (S5 Fig) and a representative dilution is shown in this figure. *PMR1 pmc1-Δ* and *pmr1-Δ pmc1-Δ*, the 1MA77/12/GAP2-Δpmc strain with or without the *PMR1-*containing plasmid, respectively; *PMR1 PMC1* and *pmr1-Δ PMC1*, the 1MA77/12/GAP2 strain with or without the *PMR1-*containing plasmid, respectively.

In *S*. *cerevisiae* inactivation of the vacuolar Ca^2+^ ATPase Pmc1 disturbs the control of Ca^2+^ concentration in the cytosol, thus leading to inability of mutant cells to grow at high levels of Ca^2+^ in the culture medium. The Pmr1 pump of *S*. *cerevisiae* is also involved in the maintenance of low level of cytosolic Ca^2+^. Inactivation of both ion pumps is lethal due to increased cytosolic Ca^2+^ concentration [3]. Inactivation of Pmc1 in *H*. *polymorpha* also leads to sensitivity to high Ca^2+^ concentrations in culture medium [29]. Although manifestations of the *pmc1-Δ* and *pmr1-Δ* mutations in *H*. *polymorpha* are similar to those in *S*. *cerevisiae*, the role of Pmr1 in the cytosolic Ca^2+^ control and the role of Pmc1 in the secretory pathway Ca^2+^ supply in *H*. *polymorpha* remained uncertain. To resolve this uncertainty, we studied interaction of the *pmc1-Δ* and *pmr1-Δ* mutations with each other. We inactivated the *PMC1* gene in the strain disrupted for *PMR1*, which carried an autonomous *PMR1*-containing plasmid. Surprisingly, the *pmc1-Δ pmr1-Δ* double mutant was able to lose the *PMR1*-containing plasmid during growth on YPD medium, though the clones lacking the plasmid were unable to grow on SD medium (Fig 3). The effect of elevated concentration of external Mn^2+^ on growth of the *pmc1-Δ pmr1-Δ* double mutant resembled that observed in the *ret1-27 pmr1-Δ* strain, since 1–3 mM MnCl_2_ allowed the *pmc1-Δ pmr1-Δ* double mutant to grow on SD medium (Fig 3). Notably, as in the case of the *pmr1-Δ ret1-27* strain, supplementing the culture medium with 5mM CaCl_2_ also rescued growth of the *pmc1-Δ pmr1-Δ* double mutant (Fig 3). The observations that the *pmc1-Δ pmr1-Δ* double mutant is viable and that its growth defect can be rescued by elevation of Ca^2+^ concentration in culture medium indicate that, in contrast to *S*. *cerevisiae*, in *H*. *polymorpha* Pmr1 is not essentially involved in the control of concentration of cytosolic Ca^2+^. The exacerbation of dependence of the *pmr1-Δ* mutant on external Ca^2+^ and Mn^2+^ by the *PMC1* inactivation suggests a role of the vacuole in supply of the secretory organelles with Ca^2+^.

**Fig 4:**
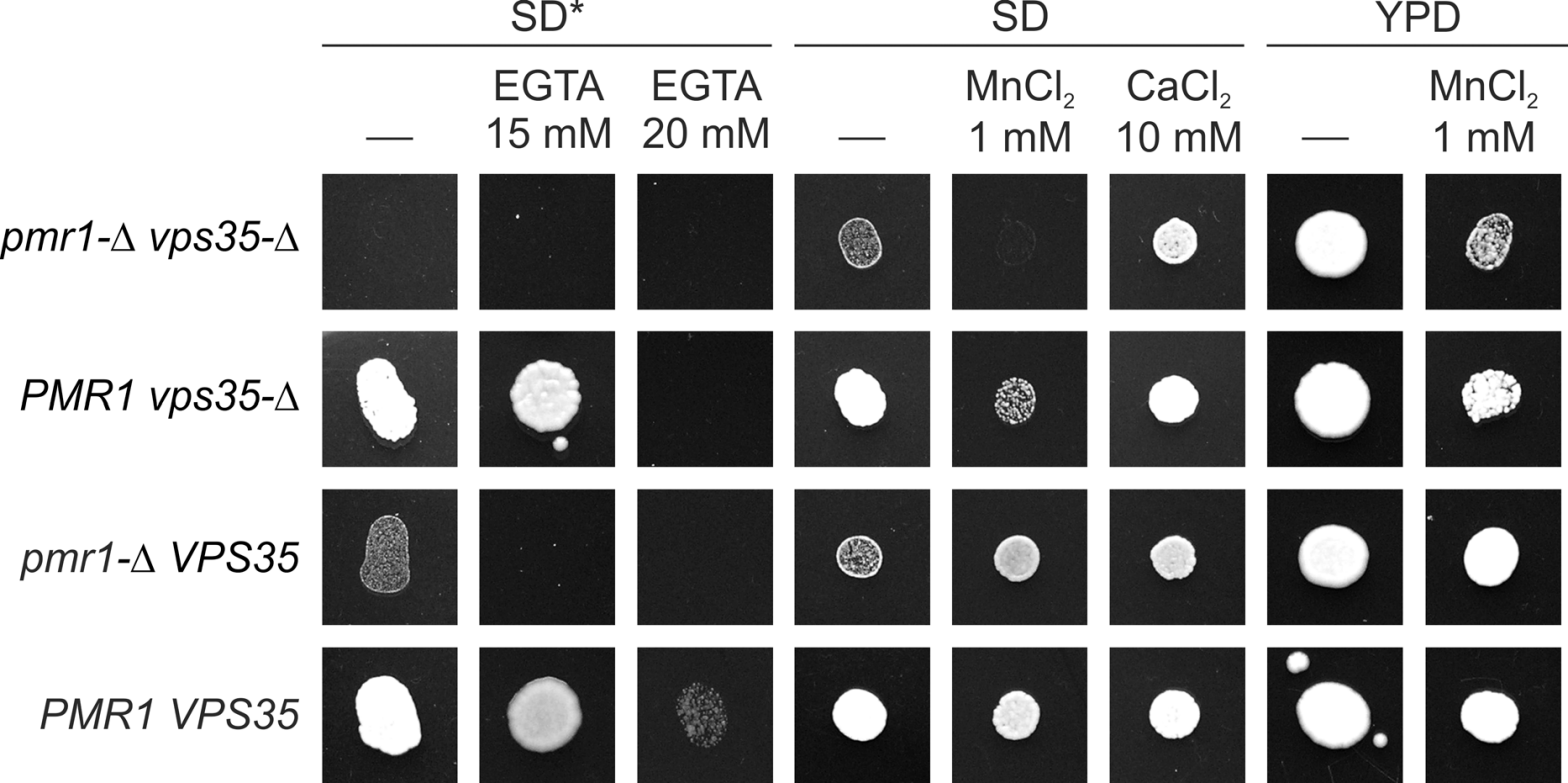
Effect of the *vps35-Δ* mutation on growth of strains with or without the *PMR1* gene. Cell suspensions with equal densities were spotted onto corresponding media and grown for 2 days. The experiment was performed serial dilutions of cell suspensions (S6 Fig) and a representative dilution is shown in this figure. *pmr1-Δ vps35-Δ*, the 1MA27/12/GP1-Δvps35 strain lacking the plasmid with *PMR1*; *vps35-Δ*, the 1MA27/12/GP1-Δvps35 strain, *pmr1-Δ VPS35*, the 1MA27/12/GP1 strain lacking the plasmid with *PMR1*; *PMR1 VPS35*, the 1MA27/12/GP1 strain.

The role of the vacuole in supplying the secretory organelles with Ca^2+^ also followed from effects of the inactivation of Vps35, which is a component of the retromer complex responsible for the retrograde trafficking from the prevacuolar compartments to the Golgi apparatus. Similarly to *ret1-27* ([18] and Fig 2), the *vps35-Δ* mutation led to hypersensitivity to Ca^2+^ shortage in culture medium and exacerbated Ca^2+^ dependence of the *pmr1-Δ* mutant (Fig 4). Indeed, the growth of the *vps35-Δ* mutant was abolished by supplementing of SD* with 20 mM EGTA, while the strain with the wild-type *VPS35* allele still could grow. The *vps35-Δ* effect on Ca^2+^ dependence of the *pmr1-Δ* mutant was less pronounced then the effect of *ret1-27*, since the *vps35-Δ pmr1-Δ* double mutant was able to grow on regular SD and YPD media. The exacerbation of the *pmr1-Δ* dependence on Ca^2+^ by the *vps35-Δ* mutation indicated involvement of pre-vacuolar compartments in a Pmr1-independent supply of the secretory organelles with Ca^2+^. At the same time the *vps35-Δ* mutation exerted hypersensitivity to Mn^2+^, which masked the ability of Mn^2+^ to suppress the growth defect caused by the *pmr1-Δ* mutation. We speculate that Vps35 is involved in degradation of the plasma membrane Mn^2+^ transporter and thus the loss of Vps35 may increase Mn^2+^ uptake.

### Defect of COPI-dependent supply of the secretory organelles with Ca^2+^ is not mediated by decreasing function of the Golgi Ca^2+^/Mn^2+^ ATPase Pmr1

**Fig 5:**
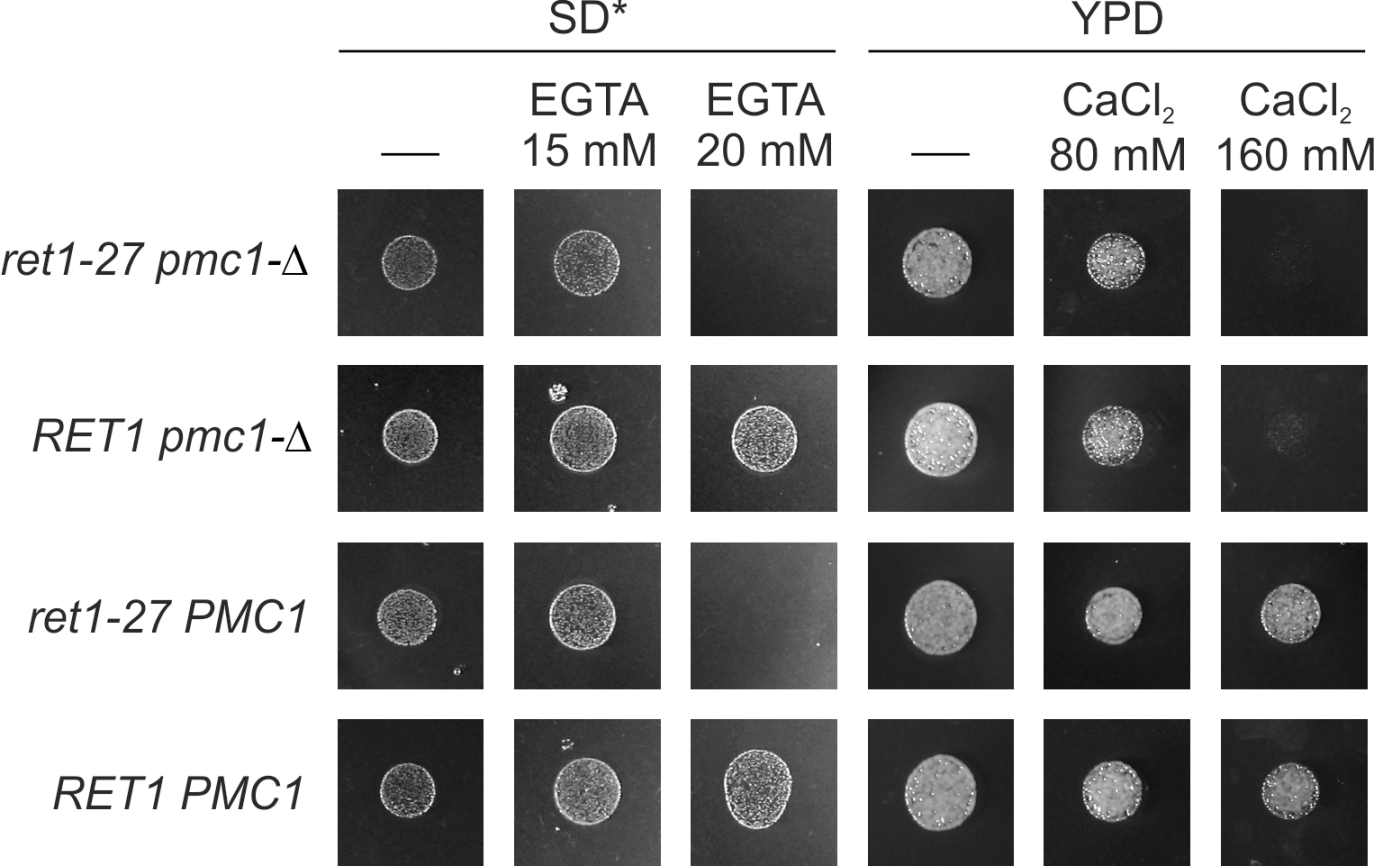
Sensitivity of the *ret1-27 pmc1-Δ* double mutant to a shortage or excess of Ca^2+^ in culture medium. Cell suspensions with equal densities were spotted onto corresponding media and grown for 2 days. Ca^2+^ shortage was achieved by addition of EGTA to SD* medium. Excess of Ca^2+^ was achieved by supplementing YPD with CaCl_2_. The experiment was repeated with serially diluted cell suspensions (S7 Fig). *ret1-27 pmc1-Δ*, the 64MA70QA-Δpmc strain; *pmc1-Δ*, the 64MA70Q-RET-Δpmc strain; *ret1-27*, the 64MA70QAL strain; WT, the 64MA70QL-RET strain.

**Fig 6:**
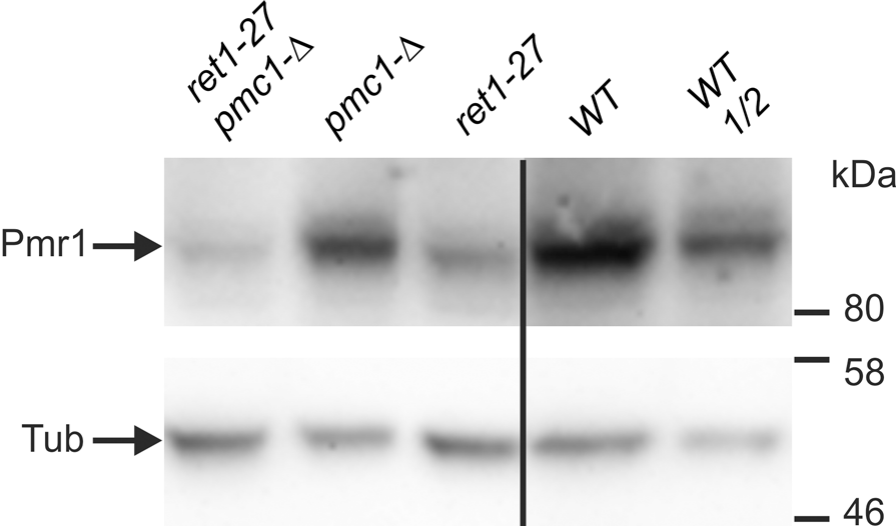
Effect of inactivation of *PMC1* on the level of Pmr1 in the *ret1-27* mutant strain and in the strain bearing the *RET1* wild-type allele. Proteins from cell lysates were resolved by SDS PAGE and transferred to nitrocellulose membrane, which was then divided in two parts at the level of the 80 kDa marker band. The upper part was stained with antiserum against *H*. *polymorpha* Pmr1, while the lower part was stained with antibody against tubulin used as a loading control. *ret1-27 pmc1-Δ*, the 64MA70QA-Δpmc strain; *pmc1-Δ*, the 64MA70Q-RET-Δpmc strain; *ret1-27*, the 64MA70QAL strain; WT and WT 1/2, undiluted and two-fold diluted sample of the 64MA70QL-RET strain, respectively.

Previously we have shown that the *ret1-27* mutation noticeably decreases the amount of the Pmr1 protein [18]. This implies that some manifestations of the *ret1-27* mutation may result from insufficient Pmr1-dependent supply of the secretory pathway with Ca^2+^ ions. Since, as it was shown above, *pmc1-Δ* exacerbates dependence of the *pmr1-Δ* mutant on external Ca^2+^ and Mn^2+^ and leads to inability to grow on synthetic medium, we expected the *pmc1-Δ* mutation to inhibit growth on synthetic medium and exacerbate dependence on external Ca^2+^ in the *ret1-27* mutant. However, this was proved to be incorrect. Specifically, *PMC1* could easily be disrupted in the *ret1-27* mutant, even though the disruptants were selected on synthetic medium. Also, the sensitivity of the *pmc1 ret1-27* double mutant to a shortage of Ca^2+^ did not differ from that of the strain bearing the *ret1-27* mutation alone, while sensitivity of this double mutant to an increased concentration of external Ca^2+^ was approximately the same as of the *pmc1* mutant (Fig 5). This indicates that the Ca^2+^ dependence of the *ret1-27* mutant is not related to insufficient function of Pmr1, since otherwise *pmc1-Δ* would exacerbate Ca^2+^ dependence of the *ret1-27* mutant. One could expect that the lack of Pmc1 should increase cytosolic Ca^2+^ concentration, which in turn can enhance expression levels of genes coding for proteins involved in the control of cytosolic Ca^2+^ concentration including Pmr1. If the *PMR1* expression was increased in response to the loss of the Pmc1 Ca^2+^ pump, it might compensate the negative effect of *ret1-27* mutation on Pmr1 level and mask exacerbation of Ca^2+^ dependence. However, this suggestion was ruled out, since no increase in the Pmr1 level in response to *PMC1* inactivation in the *ret1-27* mutant was observed (Fig 6). Moreover, the Pmr1 level was even decreased in this case, which still did not noticeably affect the *ret1-27* Ca^2+^ dependence.

### The *ret1-27* mutation causes phenotypes indicating defects in the Golgi-to-vacuole transport

**Fig 7:**
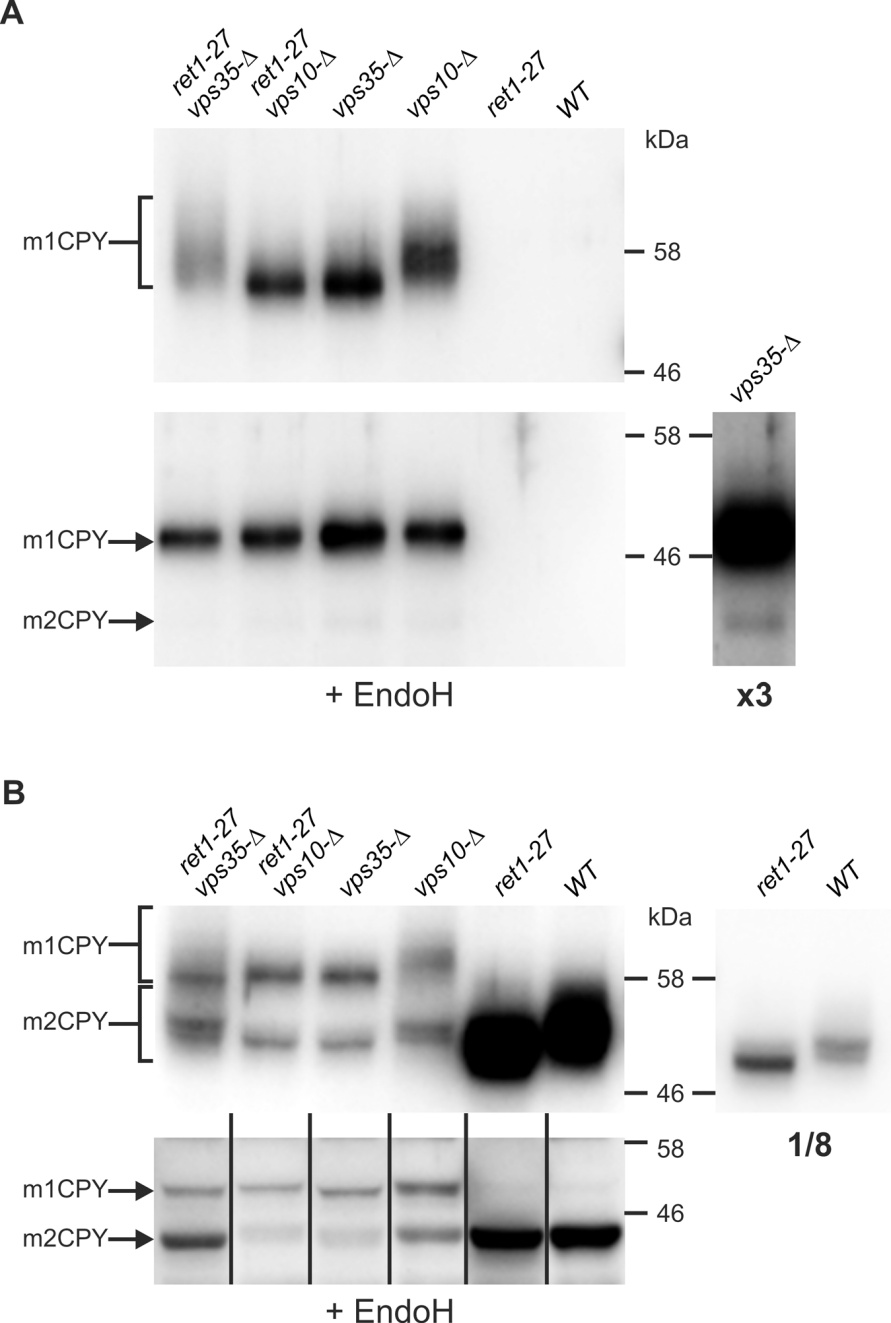
Immunoblot analysis of CPY from culture supernatants (A) and cell lysates (B). *ret1-27 vps35-Δ*, the 64MA70UA-Δvps35 strain; *ret1-27 vps10-Δ*, the 64MA70UA-Δvps10 strain; *vps35-Δ*, the 64MA70U-RET-Δvps35 strain; *vps10-Δ*, the 64MA70U-RET-Δvps10 strain; *ret1-27*, the 64MA70UAL strain; *WT*, the 64MA70UA-RET strain. +EndoH, samples treated with endoglycosidase H. X3, an overexposed image (~3-fold longer time) of the "*vps35-Δ*" lane. 1/8, an underexposed (~8-fold shorter time) image of the “*ret1-27*” and “WT” lanes.

In *S*. *cerevisiae*, some mutations in the COPI subcomplex B affect transport between the Golgi apparatus and the vacuole [22]. We suggested that *H*. *polymorpha ret1-27* also affects this pathway and leads to secretion of some vacuolar proteins, e. g. CPY. However, we did not observe any noticeable increase in the amount of CPY in the culture supernatant of the *ret1-27* mutant, while testing the *vps35-Δ* and *vps10-Δ* mutants, which have been previously shown to be defective in CPY sorting [30] showed that corresponding mutations led to accumulation of extracellular CPY and reduced its intracellular amount (Fig 7).

The *H*. *polymorpha* CPY sequence [35] contains four consensus N-glycosylation sites, one of which is located within the pro-region. In the *vps* mutants the most abundant form of extracellular CPY (Fig 7A) migrated during SDS PAGE as a dispersed band or as a smear above the 46 kDa marker band due to glycosylation, since treatment with EndoH converted it into a form migrating as a sharp ~46 kDa band. This band corresponded to CPY, which lacks only the pro-region (see Materials and Methods). We designated this form as m1CPY. Importantly, in contrast to *H*. *polymorpha*, CPY of *S*. *cerevisiae* migrates as a compact band since it possesses mature core-type uniform N-glycosides [38]. The electrophoretic pattern of *H*. *polymorpha* CPY indicated that at least one of its N-glycoside chains underwent outer chain elongation by attachment of an irregular number of mannose residues.

**Fig 8:**
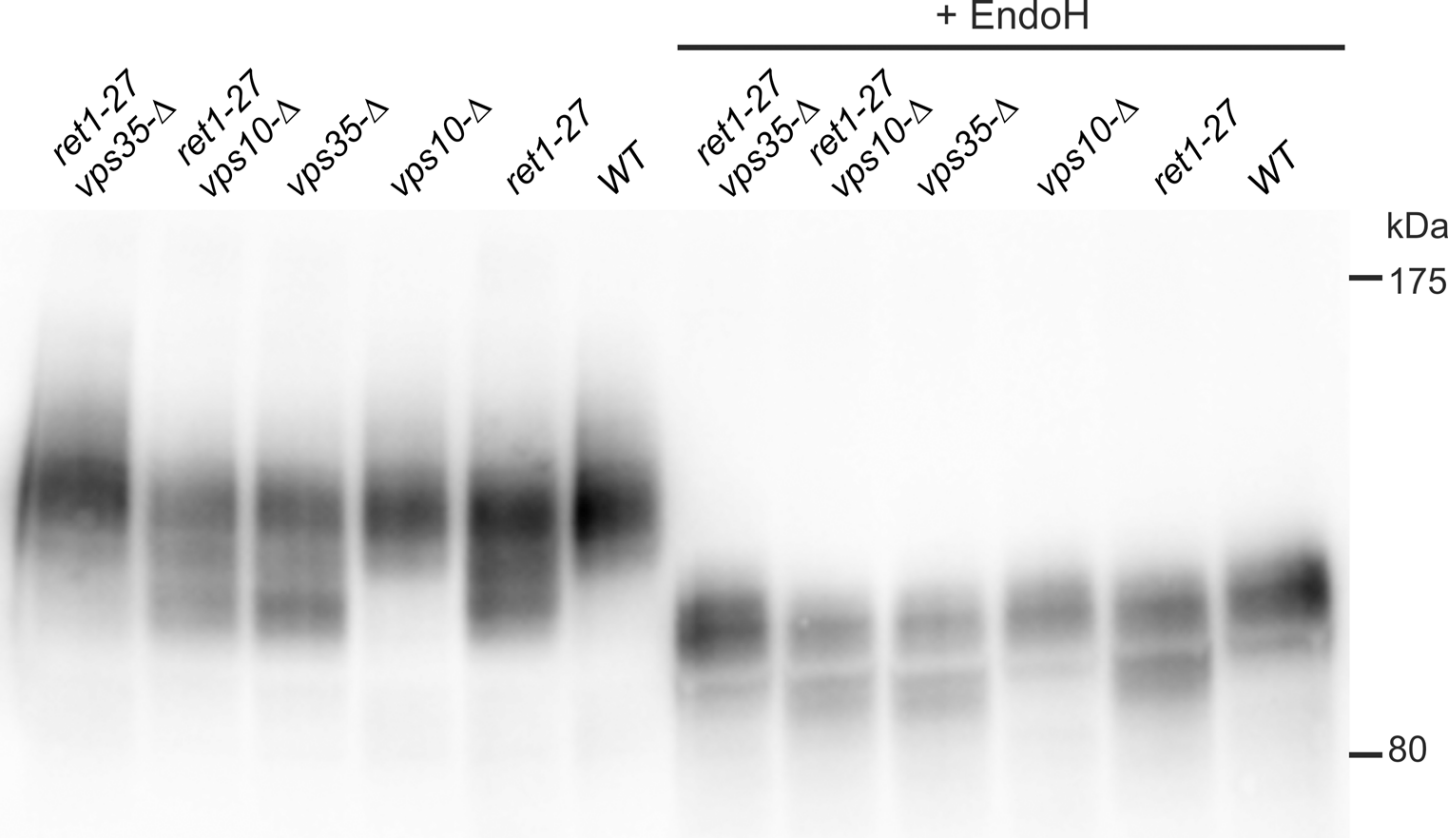
Immunoblot analysis of Gas1 from cell lysates. *ret1-27 vps35-Δ*, the 64MA70UA-Δvps35 strain; *ret1-27 vps10-Δ*, the 64MA70UA-Δvps10 strain; *vps35-Δ*, the 64MA70U-RET-Δvps35 strain; *vps10-Δ*, the 64MA70U-RET-Δvps10 strain; *ret1-27*, the 64MA70UAL strain; *WT*, the 64MA70UA-RET strain. +EndoH, samples treated with endoglycosidase H.

Glycosylation patterns of extracellular CPY depended on the *vps35-Δ*, *vps10-Δ*, and *ret1-27* mutations. Indeed, if the strains carried the wild type *RET1* allele, the *vps10-Δ* mutant secreted more extensively glycosylated CPY than the *vps35-Δ* mutant, while the *vps35-Δ* mutant secreted more extensively glycosylated enzyme than the *vps10-Δ* mutant if they carried the *ret1-27* allele (Fig 7A). The only CPY form, which was revealed in cell lysates of the strains bearing the *VPS10* and *VPS35* wild-type alleles, was the CPY fragment resulting from the additional cleavage of m1CPY (see Materials and Methods). After EndoH treatment it migrated between the 46 kDa and 30 kDa marker bands (Fig 7B). We designated this form as m2CPY. Notably, intracellular m2CPY was less glycosylated in the *ret1-27* mutant than in the wild type strain. At the same time cell lysates of the *vps10-Δ* and *vps35-Δ* mutants contained approximately the same amounts of m1CPY and m2CPY. As one could expect, the total amount of intracellular CPY in these two mutants was drastically reduced. The glycosylation of these CPY forms in the *vps35-Δ* and *vps10-Δ* mutants followed a pattern resembling that of the extracellular protein. Surprisingly, similar effects on the glycosylation pattern were observed for the cell surface protein Gas1. Particularly, it was less glycosylated in the *ret1-27* and *vps35-Δ* single mutants, while its glycosylation pattern in the strain bearing both these mutations was indistinguishable from that in the wild-type control strain. At the same time the *vps10-Δ* mutation did not noticeably affect the glycosylation pattern of Gas1 (Fig 8).

**Fig 9:**
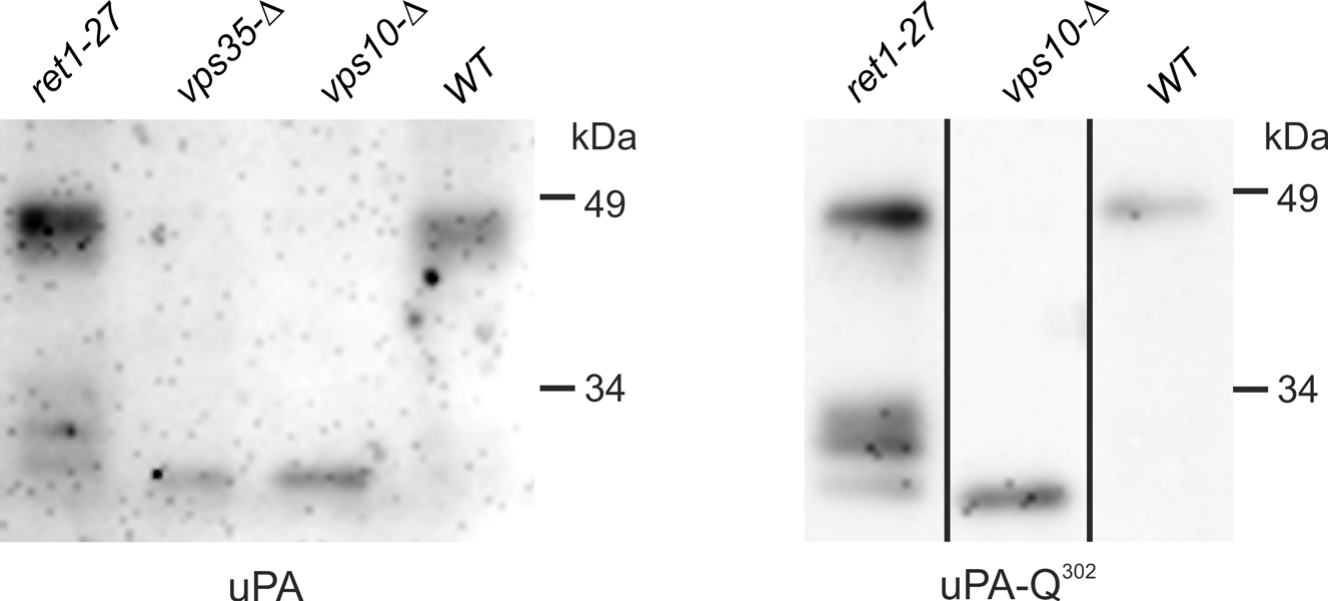
Immunoblot analysis of uPA from culture supernatants. uPA, the strains 64MA70UAL (*ret1-27*), 64MA70UA-RET (*WT*), 64MA70U-RET-Δvps10 (*vps10-Δ*), and 64MA70U-RET-Δvps35 (*vps35-Δ*), expressing the wild-type uPA; uPA-Q^302^, the strains 64MA70QAL (*ret1-27*), 64MA70QA-RET (*WT*), and 64MA70Q-RET-Δvps10 (*vps10-Δ*), expressing the unglycosylated uPA-Q^302^ mutant protein. Samples with the wild-type uPA were treated with EndoH prior to electrophoresis.

Analysis of CPY glycosylation and proteolytic processing revealed that the *ret1-27* mutation affects processes taking place downstream of the Vps10 Golgi compartment. Additional evidence for this was obtained by analyzing the proteolysis of human uPA during secretion. This protein is synthesized as a zymogen (molecular weight of polypeptide chain 46 kDa), which is activated by proteolytic cleavage of the K158-I159 peptide bond. After this cleavage uPA migrates during SDS PAGE as ~30 kDa protein. Previously we have observed that this cleavage occurs during uPA secretion by yeast cells and that defects of vacuolar protein sorting enhance the efficiency of this cleavage [30]. The *ret1-27* mutation also stimulated uPA proteolysis. This effect was even more evident for the unglycosylated mutant uPA-Q^302^. Though, in contrast to the *vps* mutants, which secreted only the 30 kDa fragment of this protein, a large portion of uPA and uPA-Q^302^ in the culture supernatant of the *ret1-27* mutant remained uncleaved and, in addition to the 30 kDa form, two slightly larger forms were also revealed (Fig 9).

**Fig 10:**
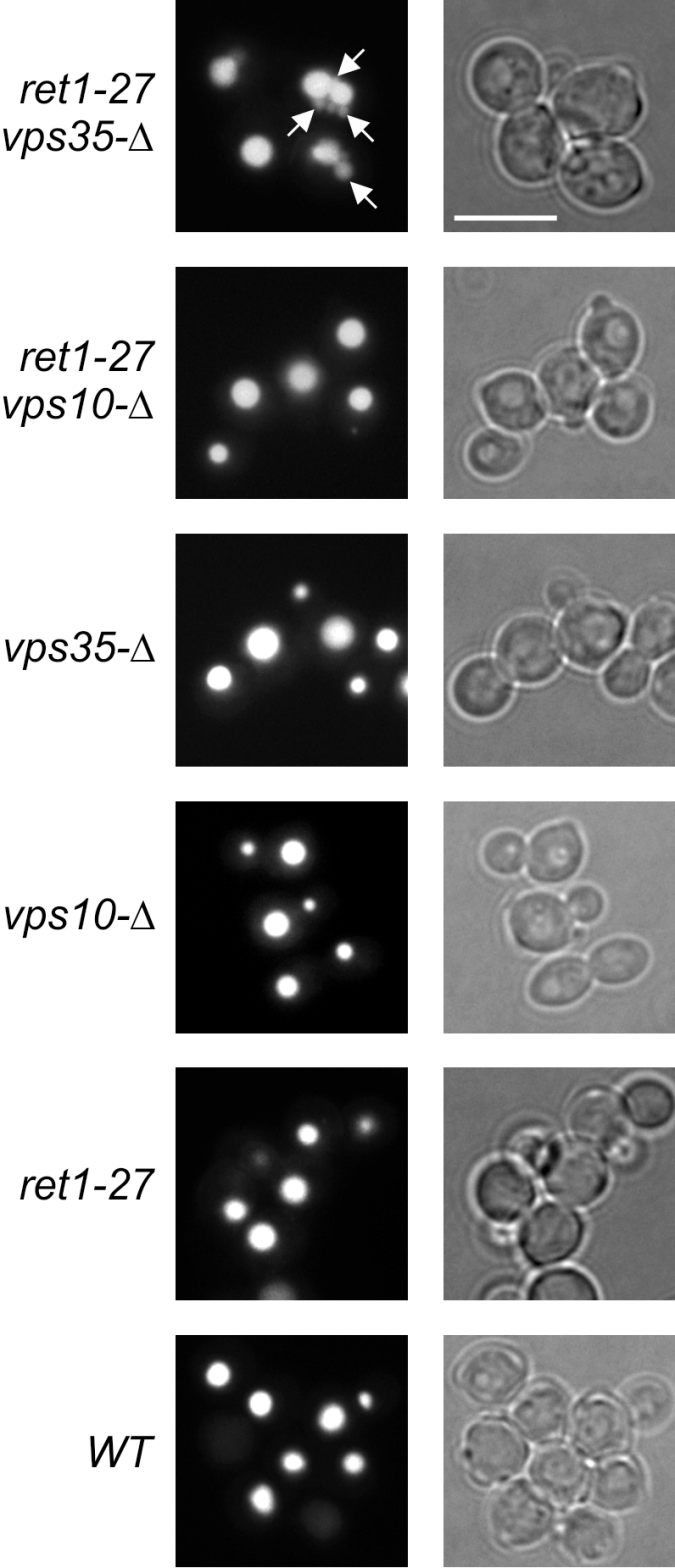
Cell Tracker Blue staining of vacuoles. *ret1-27 vps35-Δ*, the 64MA70UA-Δvps35 strain; *ret1-27 vps10-Δ*, the 64MA70UA-Δvps10 strain; *vps35-Δ*, the 64MA70U-RET-Δvps35 strain; *vps10-Δ*, the 64MA70U-RET-Δvps10 strain; *ret1-27*, the 64MA70UAL strain; *WT*, the 64MA70UA-RET strain. The white bar corresponds to 5 μm. Arrows indicate the additional compartments stained like the vacuole.

Finally we observed the effect of the *ret1-27* mutation on the vacuole morphology in the *vps35-Δ* background. The *S*. *cerevisiae vps35* mutation belongs to the A class of *vps* mutations, which do not affect morphology of the vacuole [39]. In the *H*. *polymorpha vps35-Δ* mutant, vacuole morphology was also unaltered (Fig 10). Based on the observation of increased proteolysis of uPA-Q^302^ we expected the *ret1-27* mutation to affect traffic between the secretory organelles and the vacuole. Despite this, we did not reveal any effect of this mutation alone on the vacuole morphology. However cells of the *vps35-Δ ret1-27* double mutant, in addition to the vacuole of regular morphology, possessed several smaller compartments, which were stained by a vacuole-specific dye (Fig 10).

Thus, these data indicate that similarly to the COPI sub-complex B mutations in *S*. *cerevisiae* [22], the *H*. *polymorpha ret1-27* mutation also affects traffic between the secretory organelles and the vacuole.

## Discussion

Both Ca^2+^ and Mn^2+^ ions are required for a number of processes taking place in the secretory pathway. Although the sequestration of both these ions into the secretory organelles is performed by Pmr1, which is located in the medial Golgi, the roles, which these ions play, do not overlap in most cases. For example, while Ca^2+^ is required for folding of proteins in the ER and for their binding to the vacuolar sorting receptor Vps10 [37], the functioning of mannosyltransferases in the secretory pathway depends on Mn^2+^ [40–43]. At the same time it was shown that Mn^2+^ can replace Ca^2+^ in supporting cell growth [36], though the essential cellular process, which is affected by the shortage of both Ca^2+^ and Mn^2+^ remains yet unidentified. Increased concentration of cytosolic Mn^2+^ was shown to be toxic in *S*. *cerevisiae* [11, 44, 45]. The same is true for *H*. *polymorpha* since its *pmr1-Δ* mutant is hypersensitive to an increase of Mn^2+^concentration in culture medium. Despite this, supplementing the culture medium with subtoxic concentrations of MnCl_2_ even improved growth of the *pmr1-Δ* mutant, as was observed for CaCl_2_ supplementation. This indicates that the process, in which Ca^2+^ and Mn^2+^ are interchangeable, takes place in the secretory pathway. Indeed, inactivation of Pmr1, which supplies the secretory organelles with Ca^2+^ and Mn^2+^, noticeably affects cell viability, while elevation of external concentration of either of these ions can equally alleviate the loss of Pmr1.

The *H*. *polymorpha ret1-27* mutation causes a truncation of the C-terminal domain of α-COP, which is an essential component of the COPI coat complex involved in protein and lipid traffic between the secretory organelles. Earlier it was shown that *ret1-27* causes sensitivity to Ca^2+^ shortage in culture medium and improves secretion of a heterologous protein. Since these phenotypes resemble the manifestations of the *pmr1-Δ* mutation, it was suggested that they arise due to insufficient function of Pmr1 in the *ret1-27* mutant [18]. However, here we observed that the *pmr1-Δ* and *ret1-27* mutations are synthetically lethal. Notably, the viability of this double mutant can be rescued by increasing Ca^2+^ or Mn^2+^ concentration in culture medium, which indicates that synthetic lethality was due to a shortage of these ions in the secretory organelles. Based on these data one can conclude that the *ret1-27* mutation affects Pmr1-independent supply of the secretory organelles with Ca^2+^ and Mn^2+^. If the effect of *ret1-27* on Ca^2+^ homeostasis was mediated exclusively through Pmr1, this mutation would not be able to exacerbate dependence of the *pmr1-Δ* mutant on Ca^2+^ and Mn^2+^.

In *S*. *cerevisiae*, simultaneous inactivation of the vacuolar and the Golgi Ca^2+^ ATPases is lethal due to increase in the level of cytosolic Ca^2+^ [3]. However, the *H*. *polymorpha pmr1-Δ pmc1-Δ* double mutant is able to grow on YPD medium and its growth on synthetic medium can be restored by increasing concentrations of external Ca^2+^ or Mn^2+^. Thus, the loss of the vacuolar Ca^2+^ ion pump exacerbates manifestations of the loss of the secretory pathway Ca^2+^/Mn^2+^ ATPase, but not *vice versa*. This distinguishes *H*. *polymorpha* from *S*. *cerevisiae*, in which the *pmc1 pmr1* synthetic lethality is due to exacerbation of the *pmc1* manifestation. The effect of *pmc1-Δ* on manifestations of the *pmr1-Δ* mutation observed in *H*. *polymorpha* indicates a role of the vacuole in supplying the secretory organelles with Ca^2+^ ions and agrees with the idea that the sensitivity of the *ret1-27* mutant to Ca^2+^ shortage is due to disruption of the Pmr1-independent Ca^2+^ supply of the secretory organelles. Absence of the effect of *PMC1* inactivation on manifestations of the *ret1-27* mutation supports this conclusion since *ret1-27* should block this Ca^2+^ transport pathway downstream of Pmc1, i.e. en route from the vacuole to the secretory organelles. The putative involvement of COPI dependent transport in supply of the secretory pathway with Ca^2+^ from the vacuole is supported by the implication of the COPI subcomplex B in protein transport between the Golgi apparatus and the vacuole in *S*. *cerevisiae*. Mutations in COPI subunits α, β', and ε cause defects in vacuolar protein sorting and alterations of the vacuolar morphology [22]. In the case of α-COP, only mutations in its N-terminal domain cause this effect. However the *H*. *polymorpha ret1-27* mutation, which was studied here, causes a C-terminal truncation of α-COP. Nevertheless, we have observed effects of *ret1-27* on (i) uPA-Q^302^ processing, which resemble the effect of mutations disturbing vacuolar protein sorting, on (ii) glycosylation of the vacuolar enzyme CPY, as well as (iii) on vacuolar morphology in the *vps35-Δ* background.

CPY is sorted out of the proteins, which are transported to the plasma membrane, by the Vps10 vacuolar sorting receptor. According to studies performed in *S*. *cerevisiae*, this occurs at the most trans-Golgi cisternae [8]. However in *H*. *polymorpha* we observed that CPY receives additional glycosylation when it is secreted in the absence of the Vps10 receptor, indicating that it becomes more exposed to glycosylation enzymes. This could be due to its passage through additional compartments where glycosylation occurs, or due to longer retention in the Golgi apparatus. Interestingly, inactivation of Vps35, which is responsible for retrieval of Vps10 from endosomes, leads to secretion of less glycosylated CPY than inactivation of Vps10 per se, while the *ret1-27* mutation inverses effects of these mutations on CPY glycosylation. It is worth to note that the *ret1-27* mutation decreases intracellular CPY glycosylation in the absence of vacuolar protein sorting defects as well. At the same time the effects of *ret1-27* and *vps35-Δ* mutations on the glycosylation pattern of the vacuolar protein CPY and of the cell surface protein Gas1 were very similar. Individually, each of these mutations decreased Gas1 glycosylation, while their interaction returned it to the wild-type pattern. This indicates that *ret1-27* and *vps35-Δ* affect protein glycosylation *via* different, though interacting, mechanisms. Importantly, the interaction of these mutations was also highlighted by their synthetic effect on the vacuolar morphology.

Involvement of the COPI components including α-COP in the membrane traffic from endosomes [22] can be the reason for the effect of *ret1-27* on Ca^2+^ homeostasis. Endosomes may receive Ca^2+^ from the environment or from the vacuole and then it can be transported to the secretory organelles with assistance of the COPI subcomplex B. The classical "cisternae maturation" model of the Golgi apparatus suggests recycling of the Golgi enzymes by COPI vesicles transporting them gradually from the later compartment to the earlier one [46]. In this case Ca^2+^ transported to the latest Golgi compartment would be gradually diluted en route to the earlier compartments. However, this problem is abolished if Ca^2+^ is transported directly to the earliest secretory compartments. This does not contradict the later revision of the "cisternae maturation" model [47].

Mn^2+^ does not necessarily follow the same route to the ER lumen as Ca^2+^, since it may be absorbed from the cytosol by Spf1, which is believed to be the ER Mn^2+^ ATPase [48]. Indeed, simultaneous inactivation of Pmr1 and Spf1 has much more pronounced defects of CPY* degradation and N-linked oligosaccharide trimming in the ER than individual inactivation of each of these proteins [49]. Inactivation of the plasma membrane high affinity Mn^2+^ transporter Smf1 is lethal in cells lacking Pmr1 [50]. This lethality can be overcome by increasing Ca^2+^ concentration in culture medium [51]. Similarly to the interactions of the *H*. *polymorpha pmr1-Δ* mutation with *ret1-27*, *pmc1-Δ* and *vps35-Δ* studied here, the interactions of *pmr1* with *spf1* and *smf1* in *S*. *cerevisiae* can be explained in terms of a requirement for Ca^2+^ and Mn^2+^ in a life-essential process in the secretory organelles, in which these ions are interchangeable.

## Supporting Information

S1 Fig Electrophoresis of chromosomal DNA of the MC39 strain lacking the *PMR1*-containing plasmid (*pmr1-*Δ *ret1-27*). The MC39 cells lacking the *PMR1*-containing plasmid were grown in YPD supplemented with 10 mM CaCl_2_, spun down, resuspended in regular YPD medium and incubated for the indicated time. Chromosomal DNA samples of the MC39 bearing the *PMR1*-containing plasmid (*ret1-27*) and 1MA77/12 (*pmr1-*Δ) strains grown in regular YPD were used as a control.
(PDF)

S2 Fig Effect of media composition on growth of the *pmr1-Δ* mutant (A) and on sensitivity of *H*. *polymorpha* to MnCl_2_ (B). Cell suspensions with equal densities were serially diluted (10-fold) and spotted onto corresponding media. *pmr1-Δ*, a subclone of the 1MA27/12/GP1 strain lacking the *PMR1* containing plasmid; *PMR1*, 1MA27/12/GP1 strain. Only two representative dilutions are shown in the panel B.
(PDF)

S3 Fig Growth of the *pmr1-Δ* and *ret1-27* mutants on SD* medium supplemented with different concentrations of MnCl_2_. Cell suspensions with equal densities were serially diluted (10-fold) and spotted onto corresponding media. Two subclones of each strain were analyzed. *pmr1-Δ*, subclone of the 1MA27/12/GP1 strain lacking the *PMR1* containing plasmid; *PMR1*, 1MA27/12/GP1 strain; *ret1-27*, 64MA70QAL strain; *RET1*, 64MA70QA-RET strain.
(PDF)

S4 Fig Rescue of growth of the *pmr1-Δ ret1-27* double mutant by CaCl_2_ and MnCl_2_. Since the *ret1-27 pmr1-Δ* double mutant was inviable on regular media, it was obtained from the MC39-MOX strain, which carried a *PMR1-*contaning plasmid. To allow the MC39-MOX and MC39-RET-MOX strains to lose the *PMR1*-contaning plasmid, they were streaked onto a YPD plate supplemented with 10 mM CaCl_2_. Equal amounts of cells from single colonies obtained on this medium were suspended in sterile water and spotted onto test plates. Growth of three subclones in each case is shown in this figure. Growth of the fourth subclone is shown in Fig 2. *pmr1-Δ ret1-27*, a MC39-MOX subclone lacking the plasmid; *PMR1 ret1-27*, a MC39-MOX subclone retaining the plasmid; *pmr1-Δ RET1*, a MC39-RET-MOX subclone lacking the plasmid; *PMR1 RET1*, a MC39-RET-MOX subclone retaining the plasmid.
(PDF)

S5 Fig Rescue of growth of the *pmr1-Δ pmc1-Δ* double mutant by CaCl_2_ and MnCl_2_. Cell suspensions with equal densities were serially diluted (10-fold) and spotted onto corresponding media. Two subclones of each strain were analyzed. *PMR1 pmc1-Δ* and *pmr1-Δ pmc1-Δ*, 1MA77/12/GAP2-Δpmc strain with or without the *PMR1-*containing plasmid, respectively; *PMR1 PMC1* and *pmr1-Δ PMC1*, 1MA77/12/GAP2 strain with or without the *PMR1-*containing plasmid, respectively.
(PDF)

S6 Fig Effect of the *vps35-Δ* mutation on growth of strains with or without the *PMR1* gene. Cell suspensions with equal densities were serially diluted (10-fold) and spotted onto corresponding media. Two subclones of each strain were analyzed. *pmr1-Δ vps35-Δ*, 1MA27/12/GP1-Δvps35 strain lacking the plasmid with *PMR1*; *vps35-Δ*, 1MA27/12/GP1-Δvps35 strain, *pmr1-Δ VPS35*, 1MA27/12/GP1 strain lacking the plasmid with *PMR1*; *PMR1 VPS35*, 1MA27/12/GP1 strain.
(PDF)

S7 Fig Sensitivity of the *ret1-27 pmc1-Δ* double mutant to a shortage (achieved by addition of EGTA) or excess of Ca^2+^ in culture medium. Cell suspensions with equal densities were serially diluted (10-fold) and spotted onto corresponding media. The replication of this experiment with additional concentrations of Ca^2+^ and EGTA is shown in Fig 5. *ret1-27 pmc1-Δ*, 64MA70QA-Δpmc strain; *pmc1-Δ*, 64MA70Q-RET-Δpmc strain; *ret1-27*, 64MA70QAL strain; *RET1 PMC1*, 64MA70QL-RET strain; #1 and #2, independently obtained clones.
(PDF)
