# Supplementary material for: Genetic Evidence for the Role of the Vacuole in Supplying Secretory Organelles with Ca2+ in Hansenula polymorpha
Source: PLoS One. 2015 Dec 30;10(12):e0145915. doi: 10.1371/journal.pone.0145915 (PMC4696657; doi:10.1371/journal.pone.0145915)
Supplement: S1 Fig — The MC39 cells lacking the PMR1-containing plasmid were grown in YPD supplemented with 10 mM CaCl2, spun down, resuspended in regular YPD medium and incubated for the indicated time. Chromosomal DNA samples of the MC39 bearing the PMR1-containing plasmid (ret1-27) and 1MA77/12 (pmr1-Δ) strains grown in regular YPD were used as a control. (PDF) [file pone.0145915.s001.pdf]

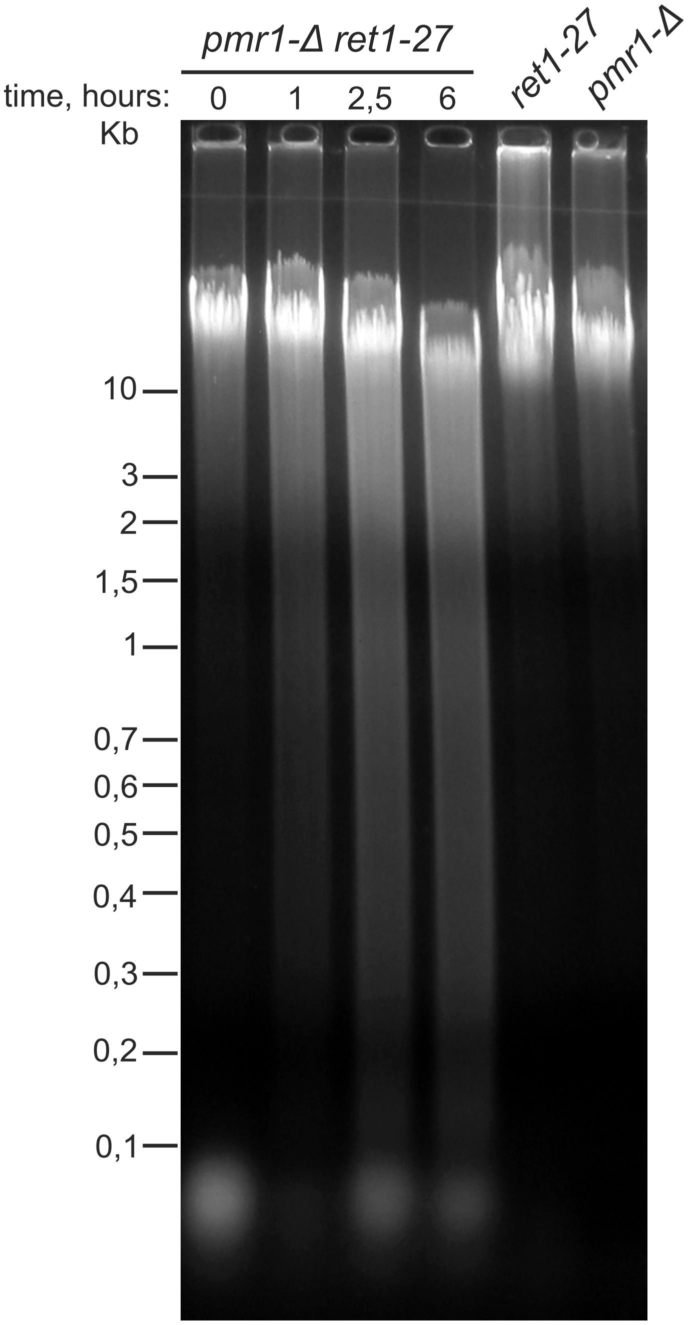

S1 Fig. Electrophoresis of chromosomal DNA of the MC39 strain lacking the *PMR1*-containing plasmid (*pmr1-*Δ *ret1-27*). The MC39 cells lacking the *PMR1*-containing plasmid were grown in YPD supplemented with 10 mM $CaCl_2$, spun down, re-suspended in regular YPD medium and incubated for the indicated time. Chromosomal DNA samples of the MC39 bearing the *PMR1*-containing plasmid (*ret1-27*) and 1MA77/12 (*pmr1-*Δ) strains grown in regular YPD were used as a control.
