# Supplementary material for: Genetic Evidence for the Role of the Vacuole in Supplying Secretory Organelles with Ca2+ in Hansenula polymorpha
Source: PLoS One. 2015 Dec 30;10(12):e0145915. doi: 10.1371/journal.pone.0145915 (PMC4696657; doi:10.1371/journal.pone.0145915)
Supplement: S2 Fig — Cell suspensions with equal densities were serially diluted (10-fold) and spotted onto corresponding media. pmr1-Δ, a subclone of the 1MA27/12/GP1 strain lacking the PMR1 containing plasmid; PMR1, 1MA27/12/GP1 strain. Only two representative dilutions are shown in the panel B. (PDF) [file pone.0145915.s002.pdf]

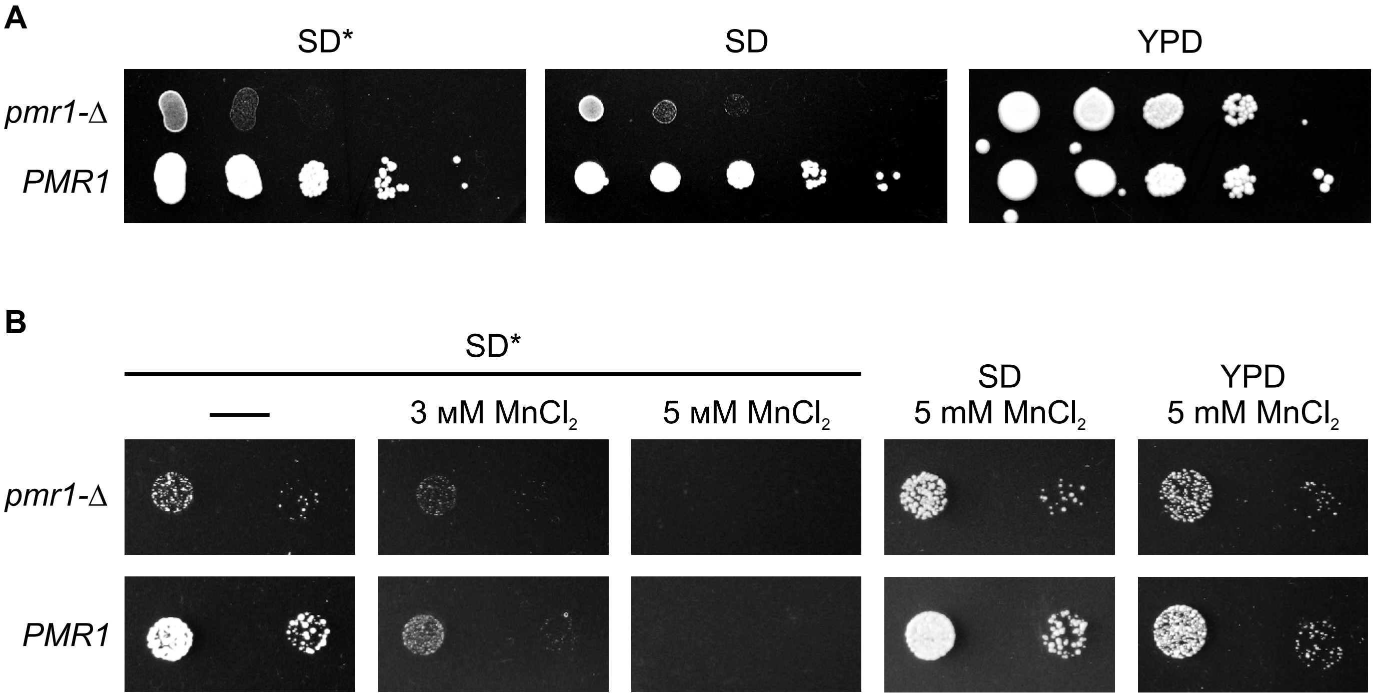

S2 Fig. Effect of media composition on growth of the *pmr1-Δ* mutant (A) and on sensitivity of *H. polymorpha* cells to $MnCl_2$ (B). Cell suspensions with equal densities were serially diluted (10-fold) and spotted onto corresponding media. *pmr1-Δ*, subclone of the 1MA27/12/GP1 strain lacking the *PMR1* containing plasmid; *PMR1*, 1MA27/12/GP1 strain. Only two most representative dilutions are shown in the panel B.
