# Supplementary material for: Genetic Evidence for the Role of the Vacuole in Supplying Secretory Organelles with Ca2+ in Hansenula polymorpha
Source: PLoS One. 2015 Dec 30;10(12):e0145915. doi: 10.1371/journal.pone.0145915 (PMC4696657; doi:10.1371/journal.pone.0145915)
Supplement: S3 Fig — Cell suspensions with equal densities were serially diluted (10-fold) and spotted onto corresponding media. Two subclones of each strain were analyzed. pmr1-Δ, subclone of the 1MA27/12/GP1 strain lacking the PMR1 containing plasmid; PMR1, 1MA27/12/GP1 strain; ret1-27, 64MA70QAL strain; RET1, 64MA70QA-RET strain. (PDF) [file pone.0145915.s003.pdf]

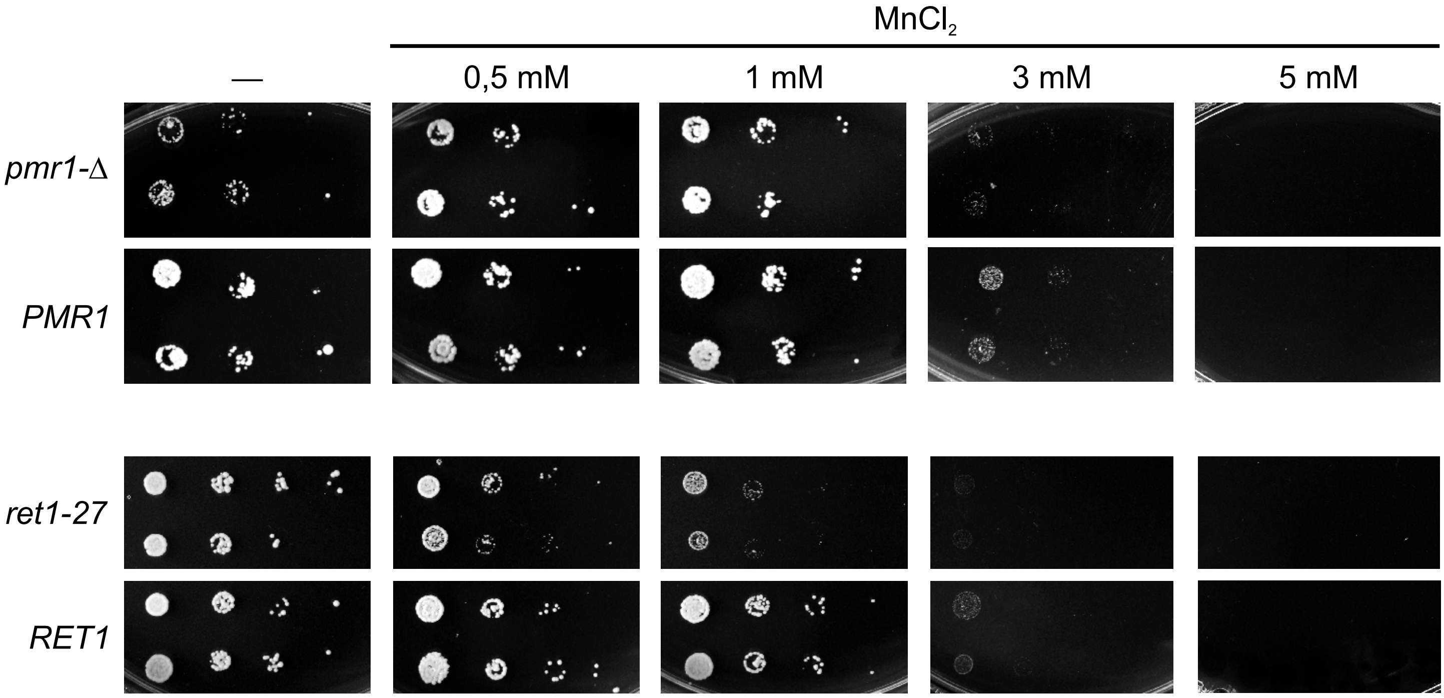


**S3 Fig. Growth of the *pmr1-Δ* and *ret1-27* mutants on SD* medium supplemented with different concentrations of $MnCl_2$.** Cell suspensions with equal densities were serially diluted (10-fold) and spotted onto corresponding media. Two subclones of each strain were analysed. *pmr1-Δ*, subclone of the 1MA27/12/GP1 strain lacking the *PMR1* containing plasmid; *PMR1*, 1MA27/12/GP1 strain; *ret1-27*, 64MA70QAL strain; *RET1*, 64MA70QA-RET strain.
