# Supplementary material for: Genetic Evidence for the Role of the Vacuole in Supplying Secretory Organelles with Ca2+ in Hansenula polymorpha
Source: PLoS One. 2015 Dec 30;10(12):e0145915. doi: 10.1371/journal.pone.0145915 (PMC4696657; doi:10.1371/journal.pone.0145915)
Supplement: S4 Fig — Since the ret1-27 pmr1-Δ double mutant was inviable on regular media, it was obtained from the MC39-MOX strain, which carried a PMR1-contaning plasmid. To allow the MC39-MOX and MC39-RET-MOX strains to lose the PMR1-contaning plasmid, they were streaked onto a YPD plate supplemented with 10 mM CaCl2. Equal amounts of cells from single colonies obtained on this medium were suspended in sterile water and spotted onto test plates. Growth of three subclones in each case is shown in this figure. Growth of the fourth subclone is shown in Fig 2. pmr1-Δ ret1-27, a MC39-MOX subclone lacking the plasmid; PMR1 ret1-27, a MC39-MOX subclone retaining the plasmid; pmr1-Δ RET1, a MC39-RET-MOX subclone lacking the plasmid; PMR1 RET1, a MC39-RET-MOX subclone retaining the plasmid. (PDF) [file pone.0145915.s004.pdf]

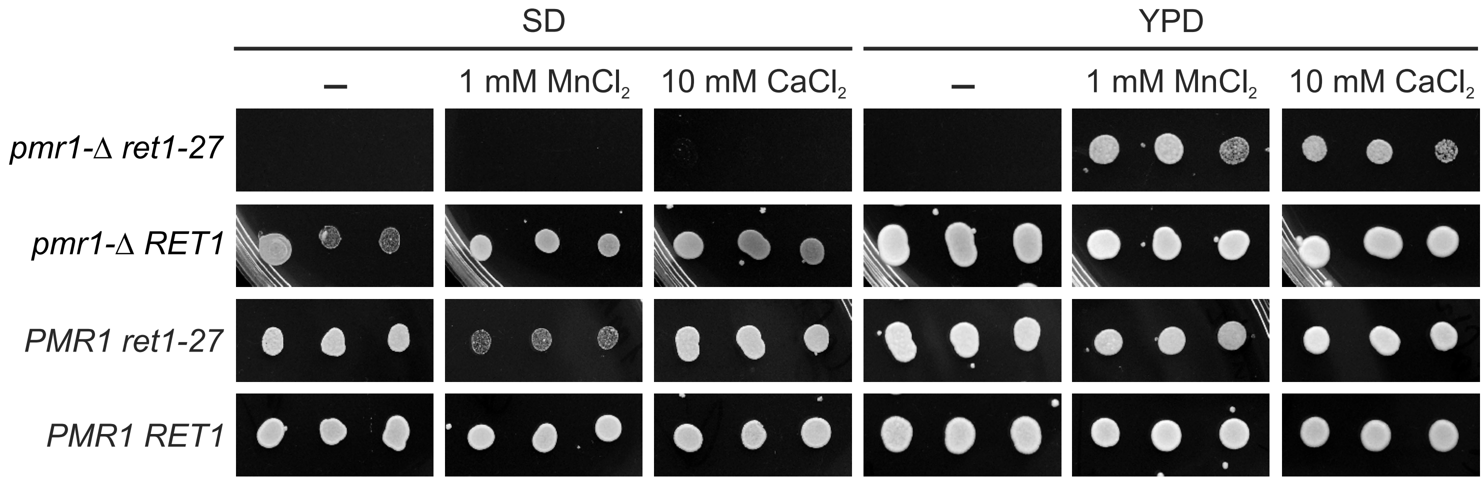

S4 Fig. Rescue of growth of the *pmr1-Δ ret1-27* double mutant by $CaCl_2$ and $MnCl_2$. Since the *ret1-27 pmr1-Δ* double mutant was inviable on regular media, it was obtained from the MC39-MOX strain, which carried a *PMR1*-contaning plasmid. To allow the MC39-MOX and MC39-RET-MOX strains to lose the *PMR1*-contaning plasmid, they were streaked onto a YPD plate supplemented with 10 mM $CaCl_2$. Equal amounts of cells from single colonies obtained on this medium were suspended in sterile water and spotted onto test plates. Growth of three subclones in each case is shown in this figure. Growth of the fourth subclone is shown in Fig. 2. *pmr1-Δ ret1-27*, a MC39-MOX subclone lacking the plasmid; *PMR1 ret1-27*, a MC39-MOX subclone retaining the plasmid; *pmr1-Δ RET1*, a MC39-RET-MOX subclone lacking the plasmid; *PMR1 RET1*, a MC39-RET-MOX subclone retaining the plasmid.
