# Supplementary material for: Genetic Evidence for the Role of the Vacuole in Supplying Secretory Organelles with Ca2+ in Hansenula polymorpha
Source: PLoS One. 2015 Dec 30;10(12):e0145915. doi: 10.1371/journal.pone.0145915 (PMC4696657; doi:10.1371/journal.pone.0145915)
Supplement: S5 Fig — Cell suspensions with equal densities were serially diluted (10-fold) and spotted onto corresponding media. Two subclones of each strain were analyzed. PMR1 pmc1-Δ and pmr1-Δ pmc1-Δ, 1MA77/12/GAP2-Δpmc strain with or without the PMR1-containing plasmid, respectively; PMR1 PMC1 and pmr1-Δ PMC1, 1MA77/12/GAP2 strain with or without the PMR1-containing plasmid, respectively. (PDF) [file pone.0145915.s005.pdf]

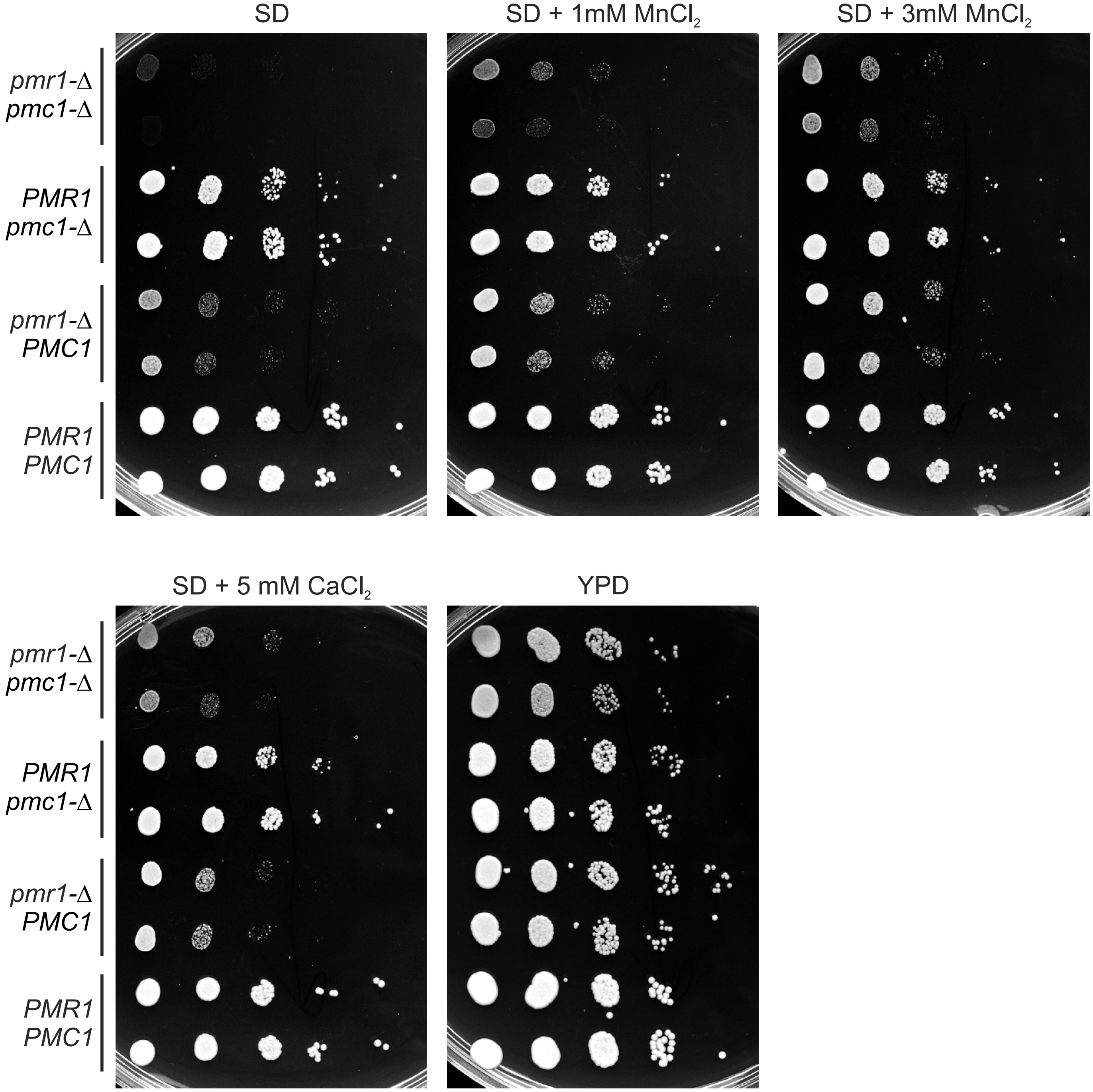

S5 Fig. Rescue of growth of the *pmr1-Δ pmc1-Δ* double mutant by $CaCl_2$ and $MnCl_2$. Cell suspensions with equal densities were serially diluted (10-fold) and spotted onto corresponding media. Two subclones of each strain were analysed. *PMR1 pmc1-Δ* and *pmr1-Δ pmc1-Δ*, 1MA77/12/GAP2-Δpmc strain with or without the *PMR1*-containing plasmid, respectively; *PMR1 PMC1* and *pmr1-Δ PMC1*, 1MA77/12/GAP2 strain with or without the *PMR1*-containing plasmid, respectively.
