# Supplementary material for: Genetic Evidence for the Role of the Vacuole in Supplying Secretory Organelles with Ca2+ in Hansenula polymorpha
Source: PLoS One. 2015 Dec 30;10(12):e0145915. doi: 10.1371/journal.pone.0145915 (PMC4696657; doi:10.1371/journal.pone.0145915)
Supplement: S6 Fig — Cell suspensions with equal densities were serially diluted (10-fold) and spotted onto corresponding media. Two subclones of each strain were analyzed. pmr1-Δ vps35-Δ, 1MA27/12/GP1-Δvps35 strain lacking the plasmid with PMR1; vps35-Δ, 1MA27/12/GP1-Δvps35 strain, pmr1-Δ VPS35, 1MA27/12/GP1 strain lacking the plasmid with PMR1; PMR1 VPS35, 1MA27/12/GP1 strain. (PDF) [file pone.0145915.s006.pdf]

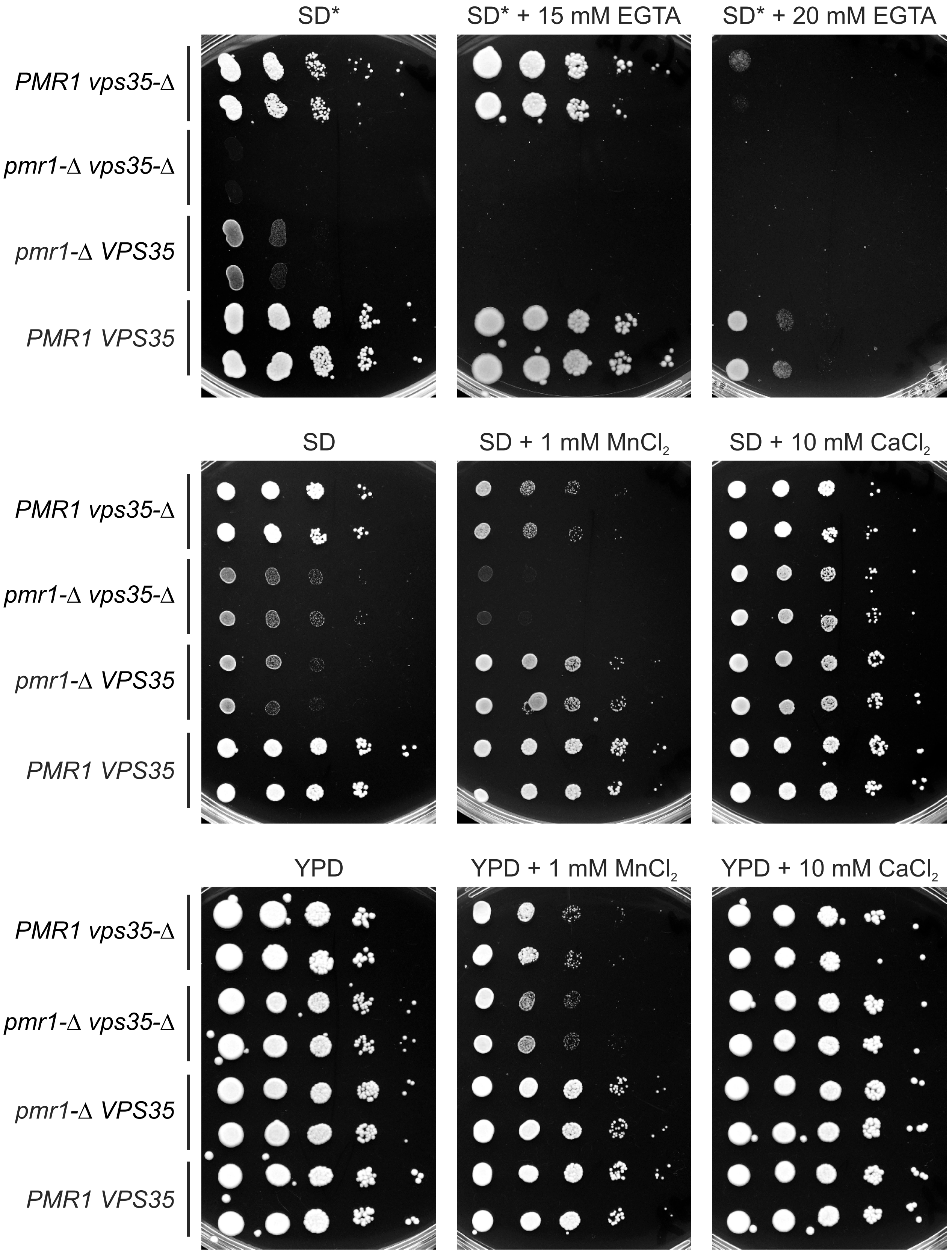

S6 Fig. Effect of the *vps35-Δ* mutation on growth of strains with or without the *PMR1* gene. Cell suspensions with equal densities were serially diluted (10-fold) and spotted onto corresponding media. Two subclones of each strain were analysed. *pmr1-Δ vps35-Δ*, 1MA27/12/GP1-Δvps35 strain lacking the plasmid with *PMR1*; *vps35-Δ*, 1MA27/12/GP1-Δvps35 strain, *pmr1-Δ VPS35*, 1MA27/12/GP1 strain lacking the plasmid with *PMR1*; *PMR1 VPS35*, 1MA27/12/GP1 strain.
