# Supplementary material for: Genetic Evidence for the Role of the Vacuole in Supplying Secretory Organelles with Ca2+ in Hansenula polymorpha
Source: PLoS One. 2015 Dec 30;10(12):e0145915. doi: 10.1371/journal.pone.0145915 (PMC4696657; doi:10.1371/journal.pone.0145915)
Supplement: S7 Fig — Cell suspensions with equal densities were serially diluted (10-fold) and spotted onto corresponding media. The replication of this experiment with additional concentrations of Ca2+ and EGTA is shown in Fig 5. ret1-27 pmc1-Δ, 64MA70QA-Δpmc strain; pmc1-Δ, 64MA70Q-RET-Δpmc strain; ret1-27, 64MA70QAL strain; RET1 PMC1, 64MA70QL-RET strain; #1 and #2, independently obtained clones. (PDF) [file pone.0145915.s007.pdf]

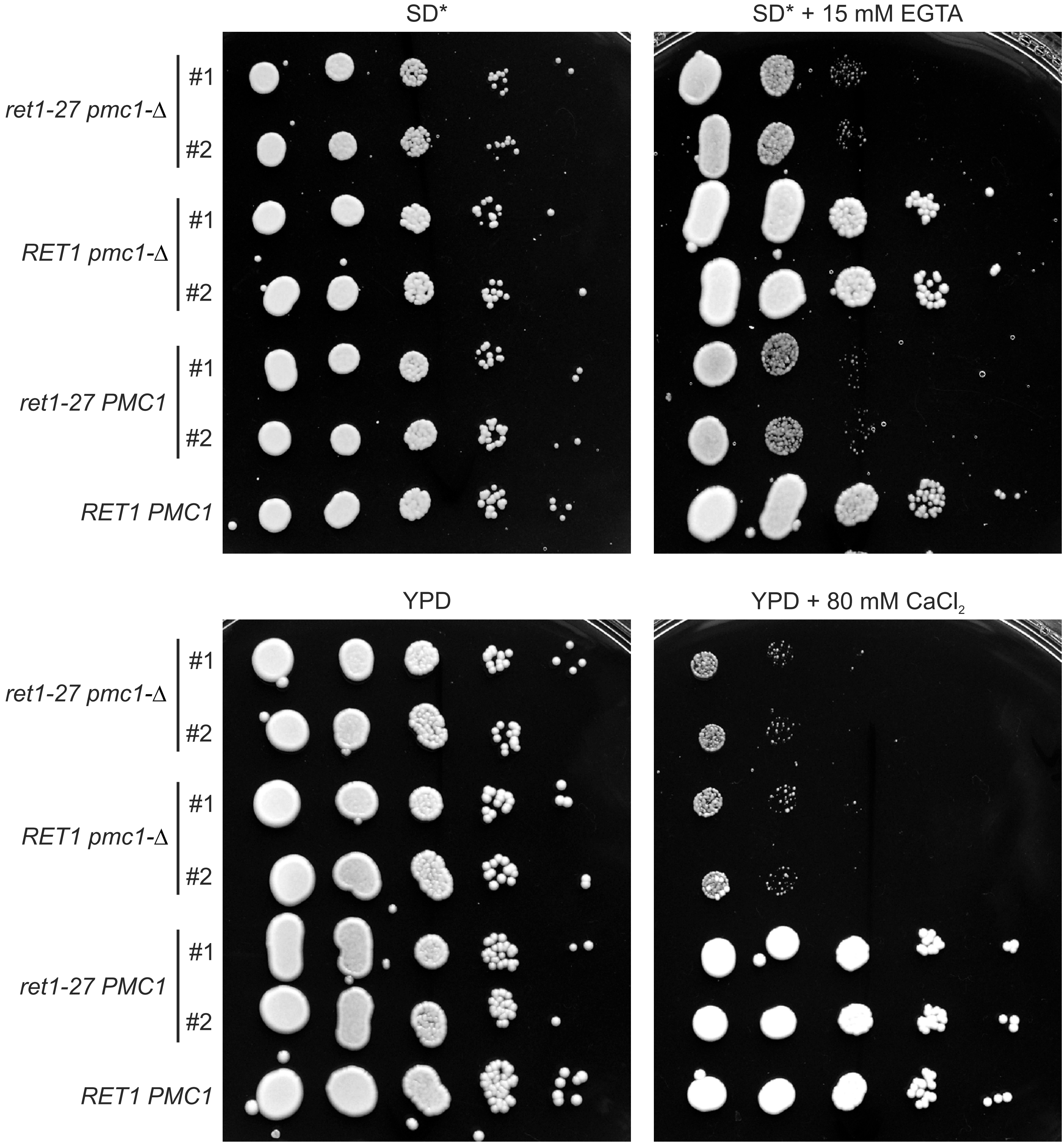

S7 Fig. Sensitivity of the *ret1-27 pmc1-Δ* double mutant to a shortage (achieved by addition of EGTA) or excess of Ca^{2+} in culture medium. Cell suspensions with equal densities were serially diluted (10-fold) and spotted onto corresponding media. The replication of this experiment with additional concentrations of Ca^{2+} and EGTA is shown in Fig. 5. *ret1-27 pmc1-Δ*, 64MA70QA-Δpmc strain; *pmc1-Δ*, 64MA70Q-RET-Δpmc strain; *ret1-27*, 64MA70QAL strain; *RET1 PMC1*, 64MA70QL-RET strain; #1 and #2, independently obtained clones.
